# Restoring circadian gene profiles in clock networks using synthetic feedback control

**DOI:** 10.1038/s41540-022-00216-x

**Published:** 2022-02-15

**Authors:** Mathias Foo, Ozgur E. Akman, Declan G. Bates

**Affiliations:** 1 School of Mechanical, Aerospace and Automotive Engineering, Coventry University, Coventry, CV1 5FB UK; 2 College of Engineering, Mathematics and Physical Science, University of Exeter, Exeter, EX4 4QF UK; 3 Warwick Integrative Synthetic Biology Centre, School of Engineering, University of Warwick, Coventry, CV4 7AL UK; 4 Present Address: School of Engineering, University of Warwick, Coventry, CV4 7AL UK

**Keywords:** Synthetic biology, Control theory

## Abstract

The circadian system—an organism’s built-in biological clock—is responsible for orchestrating biological processes to adapt to diurnal and seasonal variations. Perturbations to the circadian system (e.g., pathogen attack, sudden environmental change) often result in pathophysiological responses (e.g., jetlag in humans, stunted growth in plants, etc.) In view of this, synthetic biologists are progressively adapting the idea of employing synthetic feedback control circuits to alleviate the effects of perturbations on circadian systems. To facilitate the design of such controllers, suitable models are required. Here, we extend our recently developed model for the plant circadian clock—termed the extended S-System model—to model circadian systems across different kingdoms of life. We then use this modeling strategy to develop a design framework, based on an antithetic integral feedback (AIF) controller, to restore a gene’s circadian profile when it is subject to loss-of-function due to external perturbations. The use of the AIF controller is motivated by its recent successful experimental implementation. Our findings provide circadian biologists with a systematic and general modeling and design approach for implementing synthetic feedback control of circadian systems.

## Introduction

The daily routines of most living creatures are governed by their built-in biological clock, called the circadian system^1^. This biological clock oscillates in a quasi-sinusoidal manner with a period close to 24 h, which enables the anticipation and coordination of biological processes cued by diurnal environmental changes to happen at the most favorable time of the day. Some examples of circadian-controlled processes across different kingdoms of life include sleep/wake cycles in mammals, spore formation and release in fungi, leaf movement in plants, pupal eclosion in insects, and valve activity in bivalves (see e.g.,^2–6^), all of which are important biological functions necessary for organisms to function properly. Furthermore, many studies (see e.g.,^7–11^) have revealed a range of pathophysiological conditions associated with the disruption of the circadian rhythm (e.g., poor metabolism, psychiatric disorders, deterioration of the immune system), thereby suggesting the importance of keeping the circadian clock in a good operating condition. The general significance of circadian systems in biology is evident from the award of the 2017 Nobel Prize in Physiology or Medicine to the pioneers of circadian research^12,13^.

At the molecular level, the circadian rhythm is primarily generated through gene-protein feedback loops involving transcription and translation^14^, as well as non-transcriptional mechanisms—e.g., involving calcium^15^ and sucrose^16^ regulation. In higher organisms (e.g., mammals and plants), circadian rhythms are orchestrated by complex gene regulatory networks involving multiple clock genes. In order to gain mechanistic insights into these networks, extensive work has been undertaken by computational biologists to develop comprehensive and accurate mathematical models. These models have shown their usefulness in, for example, elucidating the effects of disruption to the plant circadian system (e.g., to plant defense^17,18^ and plant development^19^) as well as revealing the core genetic components responsible for generating oscillations in plants (e.g.,^20^).

From the perspective of synthetic biology, a disruption to the circadian system through transcription and translation mechanisms can be potentially addressed through the use of appropriate synthetic biomolecular circuits, such as those implementing feedback control. As mitigating the effects of perturbations to a system by means of feedback is an established subject of study for control engineers, synthetic biologists have started exploring the use of controller design principles to develop synthetic feedback control circuits that can be deployed to restore a disrupted natural system (see e.g.,^21–23^ and references therein).

To facilitate the systematic and robust design of a synthetic feedback control circuit, an accurate model describing the system of interest is essential. In the case of circadian systems, the most common approach used to describe transcription and translation mechanisms is Michaelis–Menten modeling with Hill-type nonlinearities (see e.g.,^24–29^). Despite the prevalence of this modeling framework in describing circadian systems, our previous work^30^ (see also Supplementary Methods) showed that when attempting to estimate Michaelis–Menten kinetic constants from temporal data, the estimated values are found to be *inconsistent*—i.e., markedly different values of the kinetic constants can reproduce the same temporal data. From the point of view of feedback control design, consistent parameter estimates are critical, since tuning of the controller design parameters for optimal performance relies heavily on these estimates. In the same work^30^, we found that a power law-based model, termed the extended S-System, does not suffer from inconsistent estimates, thereby making this modeling framework suitable for control design. In ref. ^31^, we show that this extended S-System modeling framework has comparable accuracy to equivalent Michaelis–Menten formulations in describing the plant circadian system, but with a much simpler mathematical structure.

Here, we generalize the extended S-System modeling framework to other circadian systems—namely mammals, fungi, and insects—and show how this modeling approach can be used to facilitate the design of antithetic integral feedback (AIF) controllers^32^ to restore a gene’s circadian profile when it is subject to loss-of-function due to external perturbations. The AIF controller is chosen in this study due to its recent successful experimental implementation^33^, a result that highlights its great potential for application to circadian clocks.

The application of control theory to circadian systems is not new (see e.g.,^34–38^). However, previous works typically focused on controlling the external light sources to readjust the phase of the circadian rhythms of plant or mammals that have been altered due to perturbations. These control actions are thus exerted externally (via light) and not at the molecular level. In contrast, the AIF controller considered here exerts its control action at the molecular level. Our analysis of the properties of this controller provides systematic design guidelines that should be useful to circadian biologists attempting to implement synthetic control of circadian systems.

The main contributions of this study are as follows:

- Applying the extended S-System modeling framework to non-plant circadian clocks.
- Demonstrating the applicability of the extended S-System formalism for AIF controller design.
- Providing a systematic design framework for AIF controllers in the context of circadian systems, together with a discussion of the practical implementation of the framework and some directions for future works.

## Results

### Extended S-System models for circadian systems

**Fig. 1:**
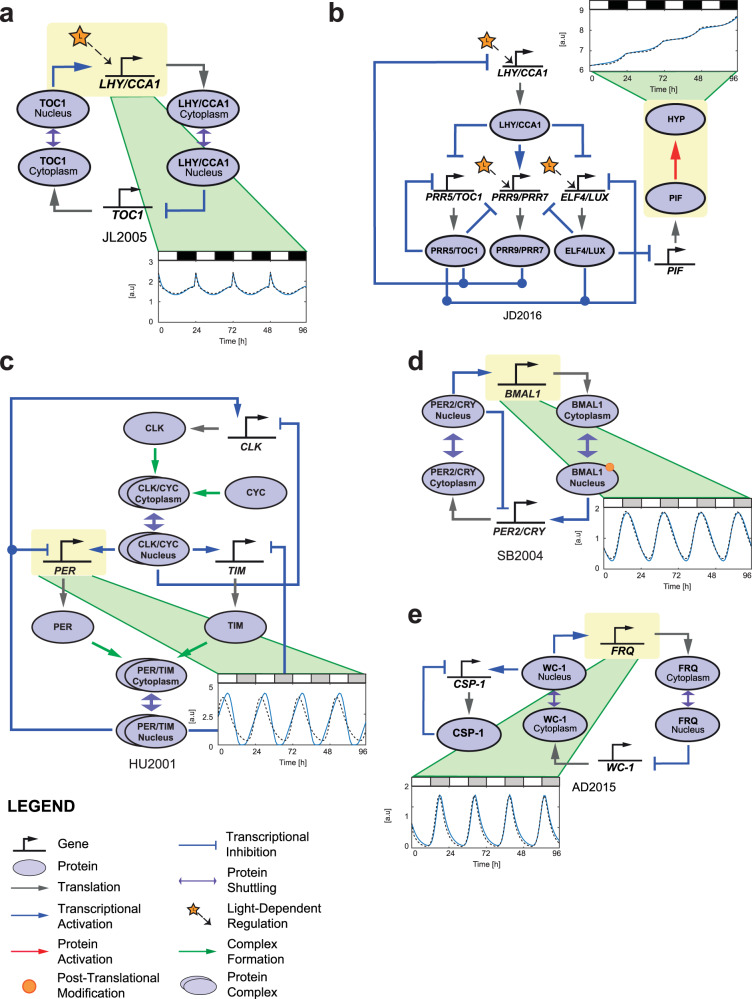
The plant, mammalian, fungal, and insect circadian clock models. **a** Plant clock, JL2005^24^. **b** Plant clock, JD2016^41^. **c** Insect clock, HU2001^25^. **d** Mammalian clock, SB2004^27^. **e** Fungal clock, AD2015^26^. For illustration, only the time series of a single gene component is presented (these components are indicated by yellow boxes). Blue solid line: Simulated time series using the standard Michaelis–Menten model from the respective literature. Black dashed line: Simulated time series using the extended S-System model. White, black, and gray rectangular boxes at the top of the figures correspond to light, dark, and subjective dark intervals, respectively. For the full comparison of all the clock genetic components, see Supplementary Figs. 2–6.

Figure 1 shows the comparison for all models between the extended S-System and Michaelis–Menten formulations, for the simulation of a representative gene component in each case (see also Supplementary Figs. 2–6 for the full comparisons). For the two plant clock models—JL2005 and JD2016—we see that the extended S-System models replicate the dynamics of the original Michaelis–Menten ones. Note that the comparison for JL2005 was also presented in our previous work^31^. The inclusion of an additional plant clock model in this study (JD2016) is principally due to this model including a downstream phenotype that we are interested in manipulating using the AIF controller. As shown in Fig. 1c–e and Supplementary Figs. 4–6, the extended S-System formulations of the mammalian, fungal, and insect circadian clocks are also able to produce similar behaviors to their Michaelis–Menten counterparts. These results confirm the capacity of the extended S-System framework to model circadian systems, beyond those of the plant clock considered previously. The extended S-System model equations for all the circadian clocks considered here can be found in Supplementary Methods, Supplementary Eqs. S1–S5. From this point onward, we will present results for two clock models only—JD2016 and AD2015. Similar results are obtained for the other three clock models (see Supplementary Methods).

### Design of AIF controllers for circadian systems

**Fig. 2:**
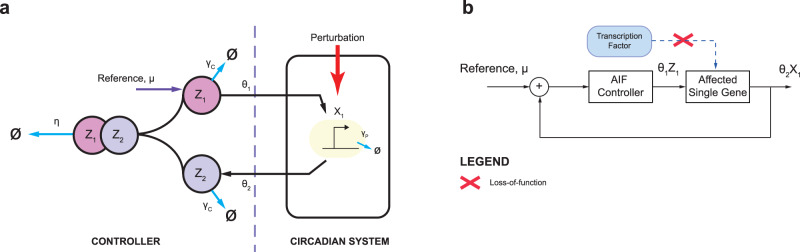
The architecture of the AIF controller. **a** The AIF controller adapted to circadian system has two control species, *Z*_1_ and *Z*_2_, where the former and the latter act as an actuator and a sensor respectively. *Z*_1_ and *Z*_2_ both degrade at the rate *γ*_*C*_. The key mechanism to achieving integral control is the sequestration of *Z*_1_ and *Z*_2_ at the rate *η*. *Z*_1_ is a product of the reference reaction, *μ*, which in turn actuates the process through *X*_1_ at the rate *θ*_1_, where the output gene *X*_1_ is sensed by *Z*_2_ at the rate *θ*_2_. **b** The closed loop configuration of the AIF controller in restoring transcription factor-driven production of an affected single gene in the circadian system.

Before proceeding with the design of the AIF controller (Fig. 2a), we first describe the control problem that will be investigated in our study. For each model, we assume that the perturbation results in the loss-of-function of a genetic component that acts as a positive regulator—these components are indicated by yellow boxes in Fig. 1. Specifically, the perturbations render the following positive regulations ineffective: upregulation of *LHY/CCA1* by TOC1 in JL2005, of HYP by PIF in JD2016, of *PER* by CLC/CYC in HU2001, of *BMAL1* by PER2/CRY in SB2004, and of *FRQ* by WC-1 in AD2015, thereby requiring a feedback controller to restore the resulting loss-of-function. Note that in HU2001, *PER* is actually regulated by two components: CLK/CYC and PER/TIM. As we are considering only the loss of positive regulation, the negative regulation of *PER* by PER/TIM is not modified.

**Fig. 3:**
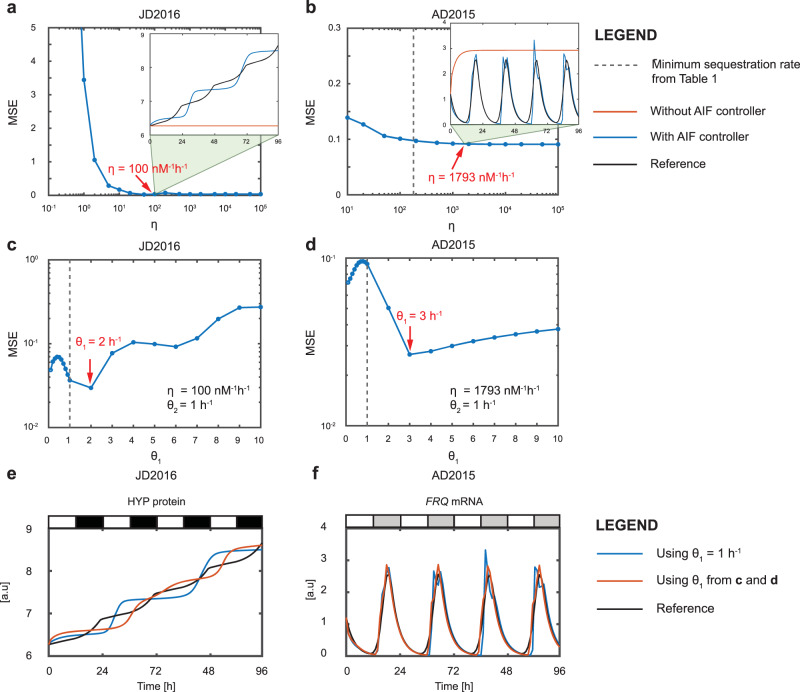
Effect of varying AIF controller parameters when controller degradation is zero. **a**, **c**, **e** Plant clock, JD2016. **b**, **d**, **f** Fungal clock, AD2015. **a**, **b** Effect of varying sequestration rate *η* on the MSE. The inset figures in **a**, **b** show the time series profiles for the reference profile and process output, with and without the use of the AIF controller. **c**, **d** Effect of varying *θ*_1_ on the MSE. **e**, **f** Time series profiles obtained using the AIF controller with different values of *θ*_1_.

Using AD2015 as an illustration, the perturbation to the positive regulation of *FRQ* by WC-1 results in the loss of a circadian profile in *FRQ* (see the red solid line in the inset of Fig. 3b). The AIF controller is therefore employed in a closed loop manner to restore the *FRQ* mRNA, as shown in Fig. 2b, where the AIF controller compares the reference *FRQ* mRNA with the output *FRQ* mRNA and actuates the correct control signal to the affected regulation such that the desired *FRQ* mRNA can be recovered.

There are four parameters that govern the dynamics of the AIF controller (see Eq. 7 in the “Methods” section). These are the sequestration rate *η*, the controller actuation rate *θ*_1_, the controller sensing rate *θ*_2_, and the controller degradation rate *γ*_*C*_. Following^39,40^, we shall first consider the case of no controller degradation, *γ*_*C*_ = 0 h^−1^, and then consider the case where *γ*_*C*_ ≠ 0 h^−1^.

#### Case when the controller degradation is zero

Among these three parameters, *θ*_1_, *θ*_2_, and *η*, the sequestration rate *η* plays an important role in determining the performance of the AIF controller. A small value of *η* yields almost no feedback between the controller and the process—hence, this will be the first parameter to be analyzed. This then begs the question of how large *η* should be. To address this, in^39,40^, Olsman et al. derived a condition on the size of *η* that ensures time scale separation between the dynamics of the system and the AIF controller. In other words, they wanted to determine whether a large value of *η* could affect the stability of the AIF controller. In their derivation, they considered the case when there are two process species (i.e., *N* = 2). In our case, as we are dealing with only one species (e.g., the *FRQ* gene in AD2015), we have *N* = 1. Following the derivation in^39,40^ the condition on *η* for *N* = 1 is given by

1 $$\eta \,> >\, \overline{\eta }={\left(\frac{{\theta }_{1}{\theta }_{2}}{{\gamma }_{P}}\right)}^{2}\left(\frac{1}{\mu }\right).$$

For the details of the derivation of Eq. 1, see^39,40^.

From Eq. 1, we compute $\overline{\eta }$ using the clock model parameter values listed in Supplementary Table 2 (JL2005), Supplementary Table 3 (JD2016), Supplementary Table 4 (SB2005), Supplementary Table 5 (AD2015), and Supplementary Table 6 (HU2001), together with their associated reference signal *μ* in each case. As the effect of the sequestration rate *η* on the AIF controller is our main interest, following the values used in refs. ^39,40^, we set *θ*_1_ = *θ*_2_ = 1 h^−1^.

#### Effect of varying sequestration rate *η*

For the plant clock model JL2005, the reference signal *μ* is the circadian profile of *LHY/CCA1*, which varies between 1.3920 and 2.4149 nM^−1^ h^−1^ (see Fig. 1a). For JD2016, the reference signal *μ* is the hypocotyl growth, represented by the protein HYP profile, which we consider over a 96 hour time interval, where it takes minimum and maximum values of 6.2713 and 21.4195 nM^−1^ h^−1^, respectively (see Fig. 1b). In the original model developed in^41^, the protein HYP is modeled as a strictly monotonically increasing function—i.e., with no degradation, *γ*_*P*_ = 0 h^−1^. This leads to $\bar{\eta }$ being undefined as we have a division by zero. Nonetheless, we will discuss our approach to choosing appropriate AIF controller parameters for this case later below.

For the non-plant clock models, the reference signals *μ* for HU2001, SB2004, and AD2015 are, respectively, the circadian profile of *PER* (which varies between 0.3450 and 3.8943 nM^−1^ h^−1^—see Fig. 1c), *BMAL1* (which varies between 0.2626 and 1.8317 nM^−1^ h^−1^—see Fig. 1d) and *FRQ* (which varies between 0.0738 and 2.5583 nM^−1^ h^−1^—see Fig. 1e). In computing $\overline{\eta }$ for all the clock models (except JD2016), we set *μ* = *μ*_min_ in Eq. 1. The resulting values of $\overline{\eta }$ are shown in the rightmost column of Table 1.

**Table 1:** Computation of sequestration rate from Eq. 1 with *θ*_1_ = *θ*_2_ = 1 h^−1^.

Model	(*μ*_min_, *μ*_max_)[nM^−1^ h^−1^]	*γ*_*P*_ [h^−1^]	$\overline{\eta }={\left(\frac{{\theta }_{1}{\theta }_{2}}{{\gamma }_{P}}\right)}^{2}\left(\frac{1}{{\mu }_{\rm{min}}}\right)\left[{{{\rm{n}}}}{{{{\rm{M}}}}}^{-1}\,{{{{\rm{h}}}}}^{-1}\right]$
JL2005	(1.3920, 2.4149)	1.2875	0.4334
JD2016	(6.2713, 21.4195)	0	Undefined
HU2001	(0.3450, 3.8943)	0.5238	10.5645
SB2004	(0.2626, 1.8317)	1.5816	1.5233
AD2015	(0.0738, 2.5583)	0.2749	179.3057

Equation 1 states that the value of *η* is required to be significantly larger than that of $\overline{\eta }$. As mentioned previously, this leads to the question of how much larger *η* should be. Although, theoretically, the value of *η* can be set arbitrarily large, in practice this value is limited by biological constraints, and thus warrants active tuning. To address this issue, we consider a range of *η* values for each clock model with *θ*_1_ and *θ*_2_ fixed at the values *θ*_1_ = *θ*_2_ = 1 h^−1^. For each *η* value applied, we calculate the mean square error (MSE) of the desired reference profile and process output using

2 $$\,{{\mbox{Mean square error, (MSE)}}}\,=\frac{1}{{N}_{T}}\mathop{\sum }\limits_{t=1}^{{N}_{T}}{\left(\mu (t)-{x}_{1}(t)\right)}^{2},$$

where *N*_*T*_ = 96 is the total number of data points, *t* is the time index, *μ* is the reference profile and *x*_1_ is the output of the process. We then plot the MSE values on a logarithmic scale against *η* for all five clock models.

As shown in Fig. 3a, b and Supplementary Fig. 7, we notice that there is an exponentially decreasing trend of the MSE values as *η* increases. This trend is consistent with that observed in^39,40^. In each MSE plot, we have also indicated the value of $\overline{\eta }$ with a dotted black line (except for JD2016 for which $\overline{\eta }$ is undefined). Interestingly, while the condition in Eq. 1 states that the value of *η* should be significantly larger than that of $\overline{\eta }$, the plots shown in those figures suggest that an *η* value of around $10\overline{\eta }$ is sufficient to obtain satisfactory AIF controller performance, as the MSE values do not change much when $\eta \,>\, 10\overline{\eta }$. For JD2016, when *η* < 10 nM^−1^ h^−1^ we observe large MSE values, while for *η* ≥ 10 nM^−1^ h^−1^, the MSE values drop significantly. Given this observation, we choose *η* = 100 nM^−1^ h^−1^ in this case, which is 10 times larger than the value at the transition point, mirroring the choice of *η* in the other four clock models.

For each gene that is assumed to be affected by perturbation in the clock models of interest, the time series profiles obtained using the AIF controller with $\eta =10\overline{\eta }$ are shown as insets in the panels of Supplementary Fig. 7. From these profiles, we see that the AIF controller restores the profiles of the affected genes quite well. By contrast, when no controller is applied, the behavior of the affected genes deviates markedly from the reference profile. For JL2005, the peaks observed in the absence of the AIF controller are due to the external light cue that regulates *LHY/CCA1*, and this shows that external light alone is unable to restore the profile of *LHY/CCA1*.

However, while the AIF controller is, in general, able to successfully restore functionality in each of the clock models, there are some unwanted effects—*viz*. the presence of multiple peaks when tracking the rising phase of the *FRQ* profile in AD2015, the transient that occurs during the tracking of the falling phase of the *LHY/CCA1* profile in JL2005, the overshooting *PER* peaks in HU2001, and a large initial *BMAL1* transient in SB2004.

Moreover, for JD2016, the AIF controller is not able to track the profile of HYP properly, oscillating around the reference signal instead. This inability to track the reference signal can be understood through the analogy of tracking a ramp signal in a linear control system (see e.g.,^42^). Like the HYP profile, a ramp signal is also a strictly monotonically increasing signal. In linear control systems analysis, to properly track a ramp signal requires the transfer function of the controller and process to contain at least two integrators and at least one zero, with the closed loop poles located in the left-hand side of the Laplace plane (s-plane)—this ensures that the closed loop poles have a negative real part, yielding exponential decay of the output oscillation to the ramp reference signal (see e.g.,^42^). Without a zero, the closed loop poles will be purely imaginary, yielding an undamped system in which the output response will continue to oscillate around the ramp reference signal. For more details of this analysis see Supplementary Methods, section S1.7.

In the case of JD2016, since HYP is modeled without the degradation term, this means there is an integrator in the process, and with the AIF controller being an integral controller, this results in the combination of the controller and the process having two integrators without any zeros. With no zero in the transfer function, the controller is unable to track the desired HYP profile accurately but oscillates around it, as we observe in Fig. 3a. Despite this, the AIF controller is able to track the increasing trend of the HYP profile and its final value does not deviate too far from that of the desired HYP final value. Given that HYP is associated with hypocotyl growth, this indicates that the controller is able to ensure sustained hypocotyl growth, albeit without the exact desired growth pattern.

Having established that the sequestration rate $\eta =10\overline{\eta }$ is sufficient to realize good performance of the AIF controller, we next proceed to investigate the effect of varying *θ*_1_ and *θ*_2_—we want to determine whether these two parameters can be tuned to further improve controller performance, in the sense of yielding smaller MSE values.

#### Effect of varying controller actuation rate, *θ*_1_

First, we vary *θ*_1_ between 0.1 and 10 h^−1^ while fixing *η* and *θ*_2_ at the values $\eta =10\overline{\eta }$ and *θ*_2_ = 1 h^−1^, computing the MSE value in each case. The results are shown in Fig. 3c, d and Supplementary Fig. 8. We note that the performance of the AIF controller can be further improved by increasing the value of *θ*_1_ through 1 h^−1^ (shown by the dotted black line), as indicated by the smaller MSE values obtained. The values of *θ*_1_ yielding the smallest MSE values are marked by red arrows and these values range between 2 and 5 h^−1^ across the models. The time series profiles for the clock genes regulated by the AIF controller that are obtained with these optimal *θ*_1_ values are shown in Fig. 3e, f and Supplementary Fig. 9, in which we also include the profiles obtained with *θ*_1_ = 1 h^−1^ (blue solid line) for comparison. As can be seen in the figures, there is noticeable improvement in the AIF controller performance when the new *θ*_1_ values are used. Notably, in JD2015 the deviation from the desired HYP profile (and in particular the final value) is smaller, in AD2015 the multiple *FRQ* peaks are no longer present, although there is still an overshoot, in JL2005 the tracking of the falling phase of the *LHY/CCA1* profile is smoother, in HU2001 the overshoot peaks of *PER* have been reduced and in SB2004 the effect of the initial *BMAL1* transient is less apparent.

#### Effect of varying controller sensing rate, *θ*_2_

Before proceeding with our investigation into the effects of varying *θ*_2_, we would like to make the following remark: we expect that this parameter should be fixed at unity in order to ensure the good performance of the AIF controller. From Eqs. 7b and 7c, with *γ*_*C*_ = 0 h^−1^, at steady state, *θ*_2_*x*_1_ = *μ*. Thus in order for *x*_1_ to track *μ* properly (i.e., to have *x*_1_ = *μ*), the scaling factor *θ*_2_ must be set to unity. To illustrate this point, we vary *θ*_2_ between 0.1 and 10 h^−1^ for each model, with *η* set to the value $\eta =10\overline{\eta }$ and *θ*_1_ set to the value indicated by the red arrow in Supplementary Fig. 8. As shown in Supplementary Fig. 10, there is no improvement in the MSE value when we tune *θ*_2_ away from 1 h^−1^. In fact, for all the clock models, any variation in *θ*_2_ results in a greater than tenfold increase of the MSE value. This reiterates our point that *θ*_2_ should be fixed at unity.

Our analyses regarding AIF controller design when the controller degradation is zero can be summarized as follows: *θ*_2_ is the most sensitive parameter and must be kept close to its designated value to ensure that the AIF controller can track the reference profile accurately. The sequestration rate *η* should be chosen to be at least 10 times the value of $\overline{\eta }$ and further improvement of the AIF controller can be achieved by tuning *θ*_1_.

#### Case when the controller degradation is non-zero

Our previous analyses considered the case of zero controller degradation (i.e., *γ*_*C*_ = 0 h^−1^). In this section, we consider the case where the controller degradation is non-zero, since—as highlighted in^39,40^—this can provide further tuning of the AIF controller performance. To start our analysis, we use the AIF controller parameters listed in Supplementary Fig. 10 with *θ*_2_ = 1 h^−1^, and vary the value of *γ*_*C*_ to determine its effect on the MSE. Note that *θ*_2_ has again been fixed at unity as any deviation from this value leads to poor controller performance (Supplementary Fig. 13), which cannot be remedied by adjusting *θ*_1_ (Supplementary Fig. 14).

**Fig. 4:**
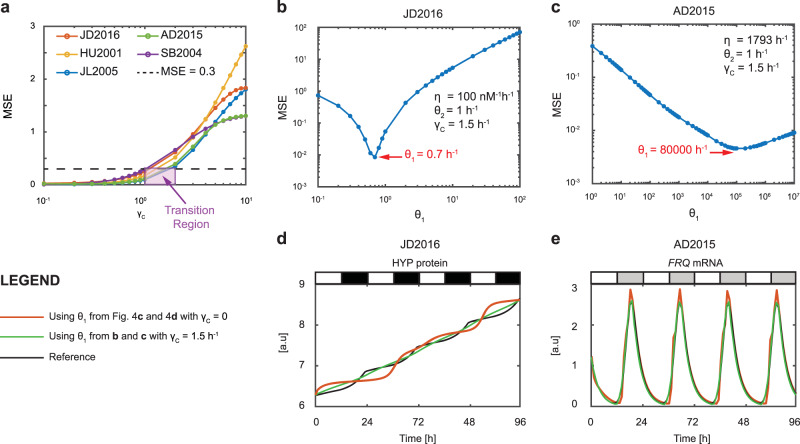
Effect of varying AIF controller parameters when controller degradation is not zero. **a** Effect of varying *γ*_*C*_ on MSE. **b**, **d** Plant clock, JD2016. **c**, **e** Fungal clock, AD2015. **b**, **c** Effect of varying *θ*_1_ on the MSE. **d**, **e** Time series profiles obtained using the AIF controller with different values of *θ*_1_.

As can be seen in Fig. 4a, all clock models exhibit the same general trend, in which the MSE value increases with *γ*_*C*_. This trend is in agreement with the findings of^39,40^, where an increase in the steady state error with *γ*_*C*_ is observed (note that as we are not tracking a constant reference signal, we use the MSE value as a proxy for the steady state error). We further observe that the MSE values increase almost linearly for *γ*_*C*_ ≤ 1 h^−1^ before increasing exponentially for *γ*_*C*_ ≥ 2 h^−1^.

Given that the MSE value increases with *γ*_*C*_, if the controller degradation rate is constrained by practical considerations to be non-zero, we next identify whether there is an acceptable MSE level associated with a particular *γ*_*C*_ value. We also investigate whether the MSE can be further decreased by tuning the other AIF controller parameters. To do this, we need to first decide on an acceptable value of the MSE (and corresponding *γ*_*C*_). With reference to Fig. 4a, we have mentioned the existence of two *γ*_*C*_ regions in which the rate at which the MSE values increase differ. In the region *γ*_*C*_ ≤ 1 h^−1^, the MSE values increase linearly, while in the region *γ*_*C*_ ≥ 2 h^−1^, the MSE values increase exponentially. At the transition region (highlighted as a purple box) 1 < *γ*_*C*_ < 2 h^−1^, the MSE value for all the clocks is approximately 0.3, which we deem to be the acceptable value. The mid-point of this transition region is *γ*_*C*_ = 1.5 h^−1^ and this is therefore selected as the controller degradation rate *γ*_*C*_ across all clock models, which we use for our further analyses.

Recall that in our analysis of the zero degradation AIF controller, we found *θ*_2_ to be the most sensitive parameter, concluding that it should not be varied at all. We are therefore left with the choice of varying *η* and *θ*_1_. As varying *η* does not change the MSE value significantly for $\eta \,>\, 10\overline{\eta }$ (see Supplementary Fig. 7), we expect that this parameter will also affect the performance of the AIF controller minimally. Thus, we are left to consider whether tuning *θ*_1_ can further improve the AIF controller performance when the controller degradation rate is non-zero.

#### Effect of varying controller actuation rate *θ*_1_ for non-zero controller degradation

To proceed with our analysis, we fix the following AIF controller parameters: $\eta =10\overline{\eta }$, *θ*_2_ = 1 h^−1^ and *γ*_*C*_ = 1.5 h^−1^. As before, we then vary *θ*_1_ and compute the corresponding MSE values. The results are shown in Fig. 4b, c and Supplementary Fig. 11. It can be seen that the performance of the AIF controller, as quantified by the MSE value, can indeed be further improved by varying *θ*_1_. We note that JD2016 has an optimal *θ*_1_ value that is less than one, whilst the other clock models have optimal *θ*_1_ values that are greater than one (see Fig. 4b and Supplementary Fig. 11). This suggests that the estimated parameters of JD2016 may not be optimal, in the sense of minimizing the cost function given in Eq. 6. In other words, there could be another set of model parameters having similar values to the set used here that can further minimize the cost function. Nevertheless, from the point of view of AIF controller design, this is consistent with our framework, i.e., we can further improve the performance of the AIF controller by tuning *θ*_1_. This observation also highlights the added robust performance of the AIF controller when dealing with parameter uncertainty. The AIF controller ensures good performance, even when the estimated parameter values are different from the true values.

To ascertain the degree of improvement, in Fig. 4d, e and Supplementary Fig. 12 we compare the time series profiles generated for all the controller-regulated clock genes when using *θ*_1_ values from Supplementary Fig. 8 to the profiles generated when using *θ*_1_ values from Supplementary Fig. 11. With the optimized *θ*_1_ values in the case *γ*_*C*_ = 1.5 h^−1^, we see further improvement in the performance of the AIF controller compared with the case *γ*_*C*_ = 0 h^−1^. Moreover, all the aforementioned unwarranted effects observed with *γ*_*C*_ = 0 h^−1^ have been significantly reduced. Specifically, in JD2016, the deviation from the reference HYP signal has been reduced substantially, in JL2005, the AIF controller can now track the falling phase of the *LHY/CCA1* profile, whilst in HU2001, SB2004 and AD2015, the AIF controller tracks the reference profiles so well that the plots overlap and are almost indistinguishable. We remark that the reduction of the unwanted effects through tuning of *θ*_1_ is also consistent with the findings of^39,40^. Taken altogether, our analyses show that a non-zero degradation rate can lead to further improvement in the AIF controller performance when *θ*_1_ is tuned appropriately.

## Discussion

In this study, we have used our previously proposed extended S-System framework to model the circadian clocks of non-plant organisms, constructing new models of the mammalian, insect, and fungal clocks. We then showed how these models can be used to facilitate the systematic design of the AIF controllers to restore the circadian profiles of genes that suffered loss-of-function due to perturbation.

As expected, the extended S-System formulation is able to represent the non-plant circadian systems with good accuracy, as shown in Fig. 1 and Supplementary Figs. 2–6. Furthermore, the corresponding estimated values of the *g*_*i*,*j*_ exponents in these models—in which positive/negative exponents represent gene activation/inhibition, respectively—are consistent with the architecture of the model used to generate the synthetic data to which it is fitted. The results presented here thus confirm the extended S-System framework as a viable and general method for quantitatively modeling circadian networks across multiple organisms.

The AIF controller in this study is designed on the basis of results from^39,40^, but here we derive the conditions for the case of single process species (i.e., *N* = 1) to cater for the application to circadian systems. There are four parameters governing the behavior of the AIF controller: the controller sequestration rate *η*, the controller actuation rate *θ*_1_, the controller sensing rate *θ*_2_, and the controller degradation rate *γ*_*C*_. We have provided a systematic analysis of the effect of each of those parameters on the AIF controller performance. Central to the overall design strategy is Eq. 1 which is derived in^39,40^, which establishes the initial design constraints.

Equation 1 establishes the plausibility condition on the choice of the sequestration rate, in the sense of stating that a rate sufficiently larger than $\overline{\eta }$ will guarantee good control. However, in practice, we do not have the luxury to choose an arbitrarily large sequestration rate as we are constrained by biological feasibility. To establish a practical sequestration rate, we found a rate $\eta =10\overline{\eta }$ was sufficient to achieve good performance of the AIF controller for each of the five clock models considered. This *η* value ranges from 4.3 to 1793 nM^−1^ h^−1^ (or 0.07 to 29.88 nM^−1^ min^−1^) across the models. These rates lie within the feasible values reported in the experimental literature, i.e., between 0.072 and 0.96 nM^−1^min^−1^ for rates (see Table SII of^43^) and up to ≈ 38.4 nM^−1^ h^−1^ for in vivo rates as reported in^44^. In addition, Aoki et al.^33^ used an *η* value of 0.05 nM^−1^min^−1^ in their modeling analysis (see Supplementary Information S.1.4.4 of^33^), which is consistent with the range found in this study. Having set the condition for the sequestration rate, our subsequent analyses showed that the controller sensing rate *θ*_2_ should be fixed at 1 h^−1^, as deviations from this rate incur large mean-square errors (MSEs) between the reference signal and the AIF controller output, as shown in Supplementary Figs. 10–13.

For the controller production rate *θ*_1_, our analyses show that further improvement in the AIF controller performance can be achieved by varying this parameter. In the case where the controller degradation is zero, we found that the *θ*_1_ values that can improve performance lie between 2 and 5 h^−1^ (or 0.033 and 0.083 min^−1^), as shown in Supplementary Fig. 8. In the case where the controller degradation is non-zero, this range is between 0.7 and 80000 h^−1^ (or 0.012 and 1333.333 min^−1^), as shown in Supplementary Fig. 11. These *θ*_1_ values are within the range 0.6–6000 h^−1^ (or 0.1–100 min^−1^) mentioned in^33^, with the exception of the *θ*_1_ value 80000 h^−1^ obtained for the fungal clock model AD2015. Nevertheless, if we look at Fig. 4c, the rate *θ*_1_ = 80000 h^−1^ corresponds to an MSE value of 0.0046. If we consider an MSE value of 0.01 to be an acceptable error, based on the MSE values observed for the four other clock models, we have *θ*_1_ = 4000 h^−1^, which is within the range reported in^33^.

In our final analysis, we investigated the performance of the AIF controller when the degradation rate *γ*_*C*_ is non-zero. With non-zero degradation, we observe that the MSE value increases as *γ*_*C*_ increases. We then proceeded to investigate whether *θ*_1_ can be used to further improve the performance of the AIF controller when the degradation rate is fixed at *γ*_*C*_ = 1.5 h^−1^ (or 0.025 min^−1^). We remark that in a practical sense, this degradation rate is similar to the degradation rate of bacteria (i.e., 0.028 min^−1^), as reported in^33^. Interestingly, our analysis revealed that a substantial improvement in AIF controller performance can be achieved by tuning *θ*_1_ (see Fig. 4d, e and Supplementary Fig. 12). In particular, we note that the tracking of HYP protein in JD2016 improves considerably, compared to the case where the controller degradation is zero. To explain this improvement, we compare the two control strategies in the context of linear control theory (see Supplementary Methods section S1.7). In essence, the presence of controller degradation has transformed a pure integral controller into a phase lag controller, which is known to reduce transient effects and decrease the steady state error. The parameter *θ*_1_ is therefore analogous to the proportional gain in a phase lag controller, and tuning this gain is commonly used in linear control theory to further reduce the steady state error (see e.g.,^42,45^). The combined effect of implementing a phase lag controller (via setting *γ*_*C*_ ≠ 0) and adjusting the proportional gain (via *θ*_1_) has resulted in the considerable improvement observed for JD2016.

This substantial improvement could be attributed to the additional degree of freedom provided by having *γ*_*C*_ ≠ 0. Moreover, given that the genetic components of the circadian system are always oscillating (i.e., always out of equilibrium), our results also indicate that an ideal integrator may not be essential in achieving adaptation for a system that does not remain at equilibrium.

In summary, in designing the AIF controller for restoring a single gene’s circadian profile, the following steps are suggested:

- Step 1 : Choose a suitable model in which the equation for the process species takes the form of Eq. 7a.
- Step 2 : Calculate $\overline{\eta }$ using Eq. 1 and choose $\eta =10\overline{\eta }$.
- Step 3 : Set *θ*_2_ to unity. If this is not possible, *θ*_1_ can be fine-tuned to achieve comparable performance (Supplementary Methods, section S1.9).
- Step 4 : Select a value for the controller degradation *γ*_*C*_ that yields an acceptable MSE value.
- Step 5 : Sweep across the biologically feasible *θ*_1_ parameter range to determine the value that yields the smallest MSE.

Note that there are algorithms available that can aid the selection of optimal AIF controller parameters, in particular for Steps 4 and 5 (see e.g.,^46^).

Here, we would like to make some remarks regarding the design of an in vitro AIF controller. In practice, the controller requires knowledge of the reference oscillatory profile *μ*(*t*) in order to correctly determine the error and thus restore the disrupted circadian profile. Realizing *μ*(*t*), which is time-varying, is challenging in comparison to realizing a constant reference profile *μ*(*t*), such as that used in^33^. Nevertheless, recent advances in the field of optogenetics have provided potential methods for realizing a time-varying reference signal. Optogenetics uses the manipulation of light to enable precise timing and local control of signaling process (see^47–49^ and references therein). In particular, Jayaraman et al. were able to use blue light regulation to control bacterial gene expression in an oscillatory manner^50,51^. In the context of generating a time-varying reference profile *μ*(*t*) for AIF control as proposed in this study, a blue light pulse could be similarly manipulated to enable bacteria to produce gene expression following the desired circadian profiles of interest, and thereby act as our *μ*(*t*) signal.

We note that employing an optogenetics approach in generating the time-varying *μ*(*t*) would introduce an additional layer of complexity to the current configuration. But to the best of our knowledge, there has not yet been any approach reported in the literature regarding the generation of time-varying reference signals for synthetic feedback control applications. While our suggested approach of using optogenetics is challenging, this should provide us with the first step towards developing this form of synthetic feedback control strategy.

Given that the optogenetics approach also requires externally exerted light signals, one could suggest using these signals directly for control, following previous work (see e.g., refs. ^34–38^). However, these studies primarily focused on using such signals to realign circadian phase. What is different in our study is that we are looking at strategies to recover circadian profiles as a result of a complete loss-of-function of a particular genetic component, which cannot be easily recovered using externally exerted light signals alone (see Supplementary Fig. 7). In view of this, a different control strategy that operates at the molecular level is required.

In addition, given that optogenetics has the potential to realize a time-varying *μ*(*t*), it could be suggested to use the signal to directly regulate the target circadian genes to compensate for the loss-of-function, hence negating the need for an AIF controller at all. From the control engineering perspective, this approach is akin to model inversion open-loop control, where the control signals are specifically designed through inversion of process dynamics to ensure proper reference tracking. It is known within the control community that model inversion open-loop control can never achieve perfect inversion of the process dynamics due to the effect of intrinsic noise and model uncertainties, and thus a feedback controller is always required to address the resulting mismatch (see e.g.,^52^). Nevertheless, in the case of circadian systems, previous studies (see e.g.^53–55^) have demonstrated the robustness of circadian profiles to noise, where the desired profiles were retained through time averaging. This opens up the possibility of using the model inversion open-loop control approach instead of a more expensive and complex synthetic feedback control, which is worth exploring further as part of our future studies.

Our current analysis of the AIF controller involves a simple clock model, and it primarily operates to restore the functionality of a single gene in the clock network. Moreover, the transcription factor that loses its function has no major impact on the other genetic component of the network. In some cases, the transcription factor that loses its function may have widespread repercussion on the overall network (e.g., TOC1 in the plant clock is known to affect five other genes in addition to *LHY/CCA1*^56^). In such cases, possible strategies include designing the AIF controller to restore the transcription factor that loses its function (Supplementary Fig. 15) or reformulating the AIF control design as a Single-Input-Multi-Output (SIMO) control problem (see e.g.,^57^), which are being considered as part of our future work.

As a final remark, even though the design framework we describe here is for circadian clocks, the approach presented is potentially applicable to tracking or restoring any biological system characterized by entrainable, periodic oscillations, for which theoretical developments are garnering great interest (see e.g.,^58,59^).

## Methods

### The extended S-System modeling framework

The S-System model structure developed by Savageau has its origins in biochemical systems theory (see e.g.,^60^), in which the model structure takes the form below:

3 $$\frac{{\rm{d}}{X}_{i}}{{\rm{d}}t}={\alpha }_{i}\mathop{\prod }\limits_{j=1}^{n+m}{X}_{j}^{{g}_{i,j}}-{\beta }_{i}\mathop{\prod }\limits_{j=1}^{n+m}{X}_{j}^{{h}_{i,j}},\quad 1\le i\le n.$$

Here, the dependent variables, $\left\{{X}_{i}:1\le i\le n\right\}$ represent the biochemical species of interest, and the independent variables, $\left\{{X}_{i}:n+1\le i\le n+m\right\}$ represent forcing terms. For each dependent variable *X*_*i*_, *α*_*i*_ represents the production rate constant, *β*_*i*_ denotes the degradation rate constant, the *g*_*i*,*j*_s are the exponents associated with production processes and the *h*_*i*,*j*_s are the exponents associated with degradation processes.

To account for the light input and other gene/protein post-translational processes characteristic of circadian clocks, Eq. 3 is extended (and hereafter termed the *extended S-System*) as follows^31^:

4 $$\frac{{\rm{d}}{X}_{i}}{{\rm{d}}t}={\alpha }_{i}\mathop{\prod }\limits_{j=1}^{{n}_{i}^{P}}{\left(\mathop{\sum }\limits_{k = 1}^{n}{b}_{i,j,k}{X}_{k}\right)}^{{g}_{i,j}}-\mathop{\sum }\limits_{j=1}^{{n}_{i}^{D}}{\beta }_{i,j}{X}_{i}\left(\mathop{\prod }\limits_{k=1}^{n}{X}_{k}^{{h}_{i,j,k}}\right)+\mathop{\sum }\limits_{j=1}^{{n}_{i}^{L}}{\gamma }_{i,j,}{U}_{i,j},\quad 1\le i\le n.$$

In the above, ${n}_{i}^{P}$, ${n}_{i}^{D}$ and ${n}_{i}^{L}$ represent the number of processes associated with production, degradation, and light regulation, respectively. *α*_*i*_, *g*_*i*,*j*_, *β*_*i*,*j*_, *h*_*i*,*j*_, and *γ*_*i*,*j*_ in turn represent the production rate constant of *X*_*i*_, the exponents associated with production, the degradation/stabilization rate constants of *X*_*i*_, the exponents associated with degradation, and the strength of the light-regulated processes affecting *X*_*i*_. The *b*_*i*,*j*,*k*_s are Boolean variables that specify the particular species contributing to the light-independent production of *X*_*i*_. Each *U*_*i*,*j*_ = *U*_*i*,*j*_(*X*_1_, …, *X*_*n*_, *L*_*T*_(*t*)) represents the effect on *X*_*i*_ of processes regulated by the external light signal, *L*_*T*_(*t*). The explicit dependence of *U*_*i*,*j*_ on $\left\{{X}_{1},\ldots ,{X}_{n}\right\}$ indicates that in addition to taking into account direct regulation by *L*_*T*_(*t*), the extended S-System model also encompasses the effects of light-regulated gene/protein expression and protein complexes. The light input *L*_*T*_(*t*) is modeled as a periodic square wave that alternates between 0 and 1, with *t* = 0 taken to correspond to dawn. With that, *L*_*T*_(*t*) has the form

5 $${L}_{T}(t)=\left\{\begin{array}{ll}1\quad &\,{{\mbox{if}}}\,0\le t\,{{\mbox{mod}}}\,24 \,<\, {P}_{H},\\ 0\quad &\,{{\mbox{otherwise,}}}\,\end{array}\right.$$

where *P*_*H*_ denotes the photoperiod (the length of the light interval). Accordingly, *P*_*H*_ = 0 corresponds to constant dark (DD) while *P*_*H*_ = 24 corresponds to constant light (LL). A symmetric light–dark cycle with alternating 12 h periods of light and dark (12L:12D) can be generated by setting *P*_*H*_ = 12. For more details on the development of this modeling framework, see^31^.

A notable feature of using Eq. 4 to represent transcription is that it can naturally accommodate either positive or negative regulation within the same model structure, unlike the Michaelis-Menten formalism where different nonlinear functions are required. The type of regulation is set by specifying the sign of the exponent *g*_*ij*_ in Eq. 4, where *g*_*ij*_ > 0 corresponds to positive regulation and *g*_*ij*_ < 0 corresponds to negative regulation.

The plant circadian clock models considered in this current study are JL2005^24^ and JD2016^41^. These were chosen as they represent basic and compact models of the plant clock network, respectively. The mammalian, fungal (*Neurospora*), and insect (*Drosophila*) clock models considered in this work are SB2004^27^, AD2015^26^, and HU2001^25^, respectively. Each model name is based on the initials of the first author followed by the year of publication. The equations for the models are given in Supplementary Eq. S1 (JL2005), Supplementary Eq. S2 (JD2016), Supplementary Eq. S3 (SB2004), Supplementary Eq. S4 (AD2015), and Supplementary Eq. S5 (HU2001) of the Supplementary Methods. Circuit diagrams for each of the clock models are shown in Fig. 1.

### Parameter estimation

To estimate the parameters of the extended S-System model, we follow the procedure detailed in^31^. First, for each clock model, the synthetic temporal data (hereinafter termed the *training set*) for all the genes/proteins in the clock are generated in a symmetric light–dark cycle (12L:12D) for four days using the original Michaelis–Menten model provided in the respective literature. Four days of data are generated in order to integrate out transient effects, thereby ensuring that the circadian profiles are in steady state (i.e., have converged to the limit cycle attractor). Once in steady state, the model parameters are estimated using the final two days of data. This training set is then used for parameter estimation by solving the optimization problem given by

6 $$\hat{{{\Theta }}}=\arg \mathop{\min }\limits_{{{\Theta }}}\frac{1}{{N}_{L}{N}_{G}}\mathop{\sum }\limits_{i=1}^{{N}_{G}}\mathop{\sum }\limits_{j=1}^{{N}_{L}}{\left(\frac{{X}_{i}({t}_{j})-{\hat{X}}_{i}({t}_{j},{{\Theta }})}{{M}_{i}}\right)}^{2},$$

where Θ represents the model parameters, *N*_*G*_ is the total number of genes/proteins in the circadian model, *N*_*L*_ is the number of time points in each synthetic timeseries, and *X*_*i*_(*t*_*j*_) and ${\hat{X}}_{i}({t}_{j},{{\Theta }})$ with 1 ≤ *j* ≤ *N*_*L*_ are the synthetic timeseries for the *i*th model component and the corresponding simulated timeseries obtained from the model with parameter set Θ, respectively^61,62^. Note that we normalize the fit to each model component with the maximum value of the corresponding synthetic timeseries, ${M}_{i}=\mathop{\max }\limits_{1\le j\le {N}_{L}}{X}_{i}({t}_{j})$^31^. This is to mitigate bias in the optimization, since the genes/proteins of each model have a diverse range of amplitudes. To solve Eq. 6, we use the MATLAB function fminsearch, which implements the Nelder–Mead simplex algorithm. The estimated parameters are given in Supplementary Table 2 (JL2005), Supplementary Table 3 (JD2016), Supplementary Table 4 (SB2005), Supplementary Table 5 (AD2015), and Supplementary Table 6 (HU2001).

### Antithetic integral feedback (AIF) controller

In control theory, integral feedback control is a fundamental approach for mitigating the effects of external perturbations on the functioning of a system. Integral feedback control guarantees that a system is able to return to its original pre-perturbation condition even in the continued presence of the perturbation—this is known as adaptation in the biology literature. In view of this, the synthetic biology community has proposed multiple types of biomolecular integral control to mitigate perturbations (e.g., see^21^ and references therein). However, to date, there have been few successful experimental implementations of these integral control strategies.

In this study, we focus our attention on the AIF controller that was proposed in^32^, for which a similar mechanism has been found in endogenous biological systems (e.g., sigma factor *σ*^70^ to anti-sigma factor rsd^63^). Our focus on the AIF controller is primarily motivated by its recent successful experimental implementation in living cells^33^.

The configuration of the AIF controller is shown in Fig. 2a, where the controller is shown to the left of the dashed line and the process to be controlled is shown to the right of the dashed line. In the original configuration proposed in^32^, the degradation of the controller species is assumed to be zero (i.e., *γ*_*C*_ = 0 h^−1^). In^39,40^, Olsman et al. extended the analysis by considering the case where *γ*_*C*_ ≠ 0 h^−1^ and this case is also considered in^33^. In this study, we carried out our design analysis of the AIF controller for circadian clocks using the results obtained in^39,40^, employing the same notation as those studies. For a more detailed derivation and theoretical analysis of the AIF controller, see^39,40^.

Consider the case where we have the simplest process with *N* = 1 (i.e., only one process species). Then the model of the interconnection between the AIF controller and the process to be controlled can be written as^39,40^

7a $$\frac{{\rm{d}}{x}_{1}}{{\rm{d}}t}={\theta }_{1}{z}_{1}-{\gamma }_{P}{x}_{1},$$

7b $$\frac{{\rm{d}}{z}_{1}}{{\rm{d}}t}=\mu -\eta {z}_{1}{z}_{2}-{\gamma }_{C}{z}_{1},$$

7c $$\frac{{\rm{d}}{z}_{2}}{{\rm{d}}t}={\theta }_{2}{x}_{1}-\eta {z}_{1}{z}_{2}-{\gamma }_{C}{z}_{2},$$

where *Z*_1_ and *Z*_2_ are the controller species, *X*_1_ is the process species directly interacting with the controller species, *θ*_1_ and *θ*_2_ are the production rates, *γ*_*C*_ and *γ*_*P*_ are the degradation rates for the controller and process species, respectively, *μ* is the reference signal that regulates *Z*_1_ and *η* is the sequestration (annihilation) rate. Following the standard convention, we use the uppercase and lowercase letters to denote the species and the associated variables respectively. When the controller degradation is not present (i.e., *γ*_*C*_ = 0), the *integral* representation is obtained via^32,40^:

8 $$\frac{{\rm{d}}{z}_{1}}{{\rm{d}}t}-\frac{{\rm{d}}{z}_{2}}{{\rm{d}}t}=\mu -{\theta }_{2}{x}_{1}\Rightarrow {z}_{1}-{z}_{2}=\int\limits_{0}^{t}\left(\mu -{\theta }_{2}{x}_{1}(\tau )\right){\rm{d}}\tau .$$

In the original configuration^32^, the AIF controller ensures perfect adaptation (where the output process species *X*_1_ exactly follows the desired reference signal *μ*) in the following manner. Consider the controller part of the AIF equations, i.e., Eqs. 7b and 7c, with *γ*_*C*_ = 0 h^−1^. Then at steady state (i.e., setting all the derivatives to zero); we have the output *x*_1_ = *μ*/*θ*_2_, indicating that the output converges proportionally by 1/*θ*_2_ to the reference signal. In the case where *γ*_*C*_ ≠ 0 h^−1^, while perfect adaptation cannot be achieved due to the loss of the integral representation, adaptation can still be achieved under certain conditions (see^39,40^).

The main feature of the AIF controller that enables adaptation is the sequestration of *Z*_1_ and *Z*_2_^33^. The sequestration rate *η* thus plays a vital role in determining the performance of the AIF controller^39,40^. Here, we, therefore, explored how the performance of the AIF controller was affected by changing the sequestration rate (and the other controller parameters).

## Supplementary information

Supporting Information

## Data Availability

All the MATLAB simulation codes are available and can be downloaded from https://github.com/mathiasfoo/aifcontrolcircadian.
