# Supplementary material for: Restoring circadian gene profiles in clock networks using synthetic feedback control
Source: NPJ Syst Biol Appl. 2022 Feb 15;8:7. doi: 10.1038/s41540-022-00216-x (PMC8847486; doi:10.1038/s41540-022-00216-x)
Supplement: Supplementary file 1 — Supporting Information [file 41540_2022_216_MOESM1_ESM.pdf]

Supporting Information

Restoring circadian gene profiles in clock networks using synthetic feedback control

Mathias Foo[1,†], Ozgur E. Akman[2] and Declan G. Bates[3]

[1]School of Mechanical, Aerospace and Automotive Engineering, Coventry University, Coventry CV1 5FB, UK
[2]College of Engineering, Mathematics and Physical Science, University of Exeter, Exeter, EX4 4QF, UK
[3]Warwick Integrative Synthetic Biology Centre, School of Engineering, University of Warwick, Coventry, CV4 7AL, UK
†Present address: School of Engineering, University of Warwick, Coventry, CV4 7AL, UK

Supplementary Methods

S1 Extended methods and results

In each model below, $c_i^{(m)}(t)$ and $c_i(t)$ denote the cellular concentration of the mRNA and protein of gene i, respectively. For the models where *c*ytoplasmic and *n*uclear protein are treated separately, these two forms are labelled as $c_i^{(c)}(t)$ and $c_i^{(n)}(t)$, respectively. In addition, for models where post-translational modification (*e.g.* phosphorylation) is considered, the modified protein is written as $c_i^{(*)}$. The different gene products labeled by i are listed in 1 Table. c_P or $c_P^{(n)}(t)$ denotes the light-activated PIF3-like protein introduced originally in JL2005[24] and subsequently used in all the other clock models considered in this study. The light input, $L_T(t)$ is modelled as a square wave with amplitude alternating between 0 and 1, representing dark and light, respectively (see main text). All simulation files can be downloaded from https://github.com/mathiasfoo/aifcontrolcircadian.

S1.1 JL2005

$$\begin{aligned}
\frac{dc_L^{(m)}}{dt} &= \alpha_1 c_T^{(n)g_{1,1}} - \beta_{1,1} c_L^{(m)} + \gamma_{1,1} c_P^{(n)} L_T(t),\\
\frac{dc_L^{(c)}}{dt} &= \alpha_2 c_L^{(m)g_{2,1}} - \beta_{2,1} c_L^{(c)},\\
\frac{dc_L^{(n)}}{dt} &= \alpha_3 c_L^{(c)g_{3,1}} - \beta_{3,1} c_L^{(n)},\\
\frac{dc_T^{(m)}}{dt} &= \alpha_4 c_L^{(n)g_{4,1}} - \beta_{4,1} c_T^{(m)},\\
\frac{dc_T^{(c)}}{dt} &= \alpha_5 c_T^{(m)g_{5,1}} - \beta_{5,1} c_T^{(c)},\\
\frac{dc_T^{(n)}}{dt} &= \alpha_6 c_T^{(c)g_{6,1}} - \beta_{6,1} c_T^{(n)},\\
\frac{dc_P^{(n)}}{dt} &= -\beta_{7,1} c_P^{(n)} + \gamma_{7,1}(1 - L_T(t)) + \gamma_{7,2} c_P^{(n)} L_T(t).
\end{aligned} \tag{S1}$$

S1.2 JD2016: Extended S-System formulation

$$\begin{aligned}
\frac{dc_{CL}^{(m)}}{dt} &= \alpha_1 c_{P97}^{g_{1,1}} c_{P51}^{g_{1,2}} - \beta_{1,1} c_{CL}^{(m)} + \gamma_{1,1} c_P L_T(t),\\
\frac{dc_{CL}}{dt} &= \alpha_2 c_{CL}^{(m)} - \beta_{2,1} c_{CL} + \gamma_{2,1} c_{CL}^{(m)} L_T(t),\\
\frac{dc_{P97}^{(m)}}{dt} &= \alpha_3 c_{CL}^{g_{3,1}} c_{P51}^{g_{3,2}} c_{EL}^{g_{3,3}} - \beta_{3,1} c_{P97}^{(m)} + \gamma_{3,1} c_P L_T(t),\\
\frac{dc_{P97}}{dt} &= \alpha_4 c_{P97}^{(m)} - \beta_{4,1} c_{P97} + \gamma_{4,1} c_{P97},
\end{aligned}$$

$$
\begin{aligned}
\frac{dc_{P51}^{(m)}}{dt} &= \alpha_5 c_{CL}^{g_{5,1}} c_{P51}^{g_{5,2}} - \beta_{5,1} c_{P51}, \\
\frac{dc_{P51}}{dt} &= \alpha_6 c_{P51}^{(m)} - \beta_{6,1} c_{P51} + \gamma_{6,1} c_{P51} L_T(t), \\
\frac{dc_{EL}^{(m)}}{dt} &= -\beta_{7,1} c_{EL}^{(m)} + \gamma_{7,1} c_{CL}^{g_{7,1}} c_{P51}^{g_{7,2}} c_{EL}^{g_{7,3}} L_T(t), \\
\frac{dc_{EL}}{dt} &= \alpha_8 c_{EL}^{(m)} - \beta_{8,1} c_{EL} + \gamma_{8,1} c_{EL} L, \\
\frac{dc_P}{dt} &= \alpha_9 - \beta_{9,1} c_P + \gamma_{9,1} L_T(t) + \gamma_{9,2} c_P L_T(t), \\
\frac{dc_{PIF}^{(m)}}{dt} &= \alpha_{10} c_{EL}^{g_{10,1}} - \beta_{10,1} c_{PIF}^{(m)}, \\
\frac{dc_{PIF}}{dt} &= \alpha_{11} c_{PIF}^{(m)} - \beta_{11,1} c_{PIF} + \gamma_{11,1} c_{PIF} L, \\
\frac{dc_{HYP}}{dt} &= \alpha_{12} c_{PIF}^{g_{12,1}}.
\end{aligned}
\tag{S2}
$$

S1.3 SB2004: Extended S-System formulation

$$
\begin{aligned}
\frac{dc_{PER2CRY}^{(m)}}{dt} &= \alpha_1 (c_{PER2CRY}^{(n)})^{g_{1,1}} (c_{BMAL1}^{(n)})^{g_{1,2}} - \beta_{1,1} c_{PER2CRY}^{(m)}, \\
\frac{dc_{PER2CRY}^{(c)}}{dt} &= \alpha_2 c_{PER2CRY}^{(m)} - \beta_{2,1} c_{PER2CRY}^{(c)}, \\
\frac{dc_{PER2CRY}^{(n)}}{dt} &= \alpha_3 (c_{PER2CRY}^{(c)})^{g_{3,1}} - \beta_{3,1} c_{PER2CRY}^{(n)}, \\
\frac{dc_{BMAL1}^{(m)}}{dt} &= \beta_{4,1} (c_{PER2CRY}^{(n)})^{g_{4,1}} - \beta_{4,1} c_{BMAL1}^{(m)}, \\
\frac{dc_{BMAL1}^{(c)}}{dt} &= \alpha_{5,1} (c_{BMAL1}^{(m)})^{g_{5,1}} - \beta_{5,1} c_{BMAL1}^{(c)}, \\
\frac{dc_{BMAL1}^{(n)}}{dt} &= \alpha_6 (c_{BMAL1}^{(c)})^{g_{6,1}} - \beta_{6,1} c_{BMAL1}^{(n)}
\end{aligned}
\tag{S3}
$$

S1.4 AD2015: Extended S-System formulation

$$
\begin{aligned}
\frac{dc_{FRQ}^{(m)}}{dt} &= \alpha_1 (c_{WC1}^{(n)})^{g_{1,1}} - \beta_{1,1} c_{FRQ}^{(m)}, \\
\frac{dc_{FRQ}^{(c)}}{dt} &= \alpha_2 c_{FRQ}^{(m)} - \beta_{2,1} c_{FRQ}^{(c)}, \\
\frac{dc_{FRQ}^{(n)}}{dt} &= \alpha_3 c_{FRQ}^{(c)} - \beta_{3,1} c_{FRQ}^{(n)} - \beta_{3,2} c_{FRQ}^{(n)} c_{WC1}^{(n)} + \beta_{3,3} c_{FW}^{(n)}, \\
\frac{dc_{WC1}^{(m)}}{dt} &= \beta_{4,1} c_{CSP1}^{g_{4,1}} - \beta_{4,1} c_{WC1}^{(n)}, \\
\frac{dc_{WC1}^{(c)}}{dt} &= \alpha_{5,1} (c_{WC1}^{(m)})^{g_{5,1}} (c_{FRQ}^{(c)})^{g_{5,2}} - \beta_{5,1} c_{WC1}^{(c)}, \\
\frac{dc_{WC1}^{(n)}}{dt} &= \alpha_6 c_{WC1}^{(c)} - \beta_{6,1} c_{WC1}^{(n)} - \beta_{6,2} c_{FRQ}^{(n)} c_{WC1}^{(n)} + \beta_{6,3} c_{FW}^{(n)}, \\
\frac{dc_{FW}^{(n)}}{dt} &= \alpha_7 c_{FRQ}^{(n)} c_{WC1}^{(n)} - \beta_{7,1} c_{FW}^{(n)}, \\
\frac{dc_{CSP1}^{(m)}}{dt} &= \alpha_8 (c_{WC1}^{(n)})^{g_{8,1}} (c_{CSP1})^{g_{8,2}} - \beta_{8,1} c_{CSP1}^{(m)},
\end{aligned}
$$

$$\frac{dc_{CSP1}}{dt} = \alpha_9 c_{CSP1}^{(m)} - \beta_{9,1} c_{CSP1}. \tag{S4}$$

S1.5 HU2001: Extended S-System formulation

$$\begin{aligned}
\frac{dc_{PER}^{(m)}}{dt} &= \alpha_1 (c_{CC}^{(n)})^{g_{1,1}} (c_{PT}^{(n)})^{g_{1,2}} - \beta_{1,1} c_{PER}^{(m)}, \\
\frac{dc_{PER}}{dt} &= \alpha_2 (c_{PER}^{(m)})^{g_{2,1}} - \beta_{2,1} c_{PER} - \beta_{2,2} c_{PER} c_{TIM} - \beta_{2,3} c_{PT}^{(c)}, \\
\frac{dc_{TIM}^{(m)}}{dt} &= \alpha_3 (c_{PT}^{(n)})^{g_{3,1}} (c_{CC}^{(n)})^{g_{3,2}} - \beta_{3,1} c_{TIM}^{(m)}, \\
\frac{dc_{TIM}}{dt} &= \alpha_4 (c_{TIM}^{(m)})^{g_{4,1}} - \beta_{4,1} c_{TIM} - \beta_{4,2} c_{TIM} c_{PER} - \beta_{4,3} c_{PT}^{(c)}, \\
\frac{dc_{PT}^{(c)}}{dt} &= \alpha_5 c_{PER}^{g_{5,1}} c_{TIM}^{g_{5,2}} - \beta_{5,1} c_{PT}^{(c)}, \\
\frac{dc_{PT}^{(n)}}{dt} &= \alpha_6 (c_{PT}^{(c)})^{g_{6,1}} - \beta_{6,1} c_{PT}^{(n)}, \\
\frac{dc_{CLK}^{(m)}}{dt} &= \alpha_7 (c_{CC}^{(n)})^{g_{7,1}} (c_{PT}^{(n)})^{g_{7,2}} - \beta_{7,1} c_{CLK}^{(m)}, \\
\frac{dc_{CLK}}{dt} &= \alpha_8 (c_{CLK}^{(m)})^{g_{8,1}} - \beta_{8,1} c_{CLK} - \beta_{8,2} c_{CC}^{(c)}, \\
\frac{dc_{CC}^{(c)}}{dt} &= \alpha_9 c_{CLK}^{g_{9,1}} - \beta_{9,1} c_{CC}^{(c)}, \\
\frac{dc_{CC}^{((n))}}{dt} &= \alpha_{10} (c_{CC}^{(c)})^{g_{10,1}} - \beta_{10,1} c_{CC}^{(n)}.
\end{aligned} \tag{S5}$$

S1.6 Motivation for employing the extended S-System framework

In our previous studies [SR1, SR2], we highlighted the importance of *inconsistent parameter estimates* (*i.e.* multiple optimisation runs started from different initial conditions locating different parameter values that reproduce the same data). Michaelis-Menten models yield inconsistent parameter estimates – we illustrate here how this can pose a serious issue when designing and applying the AIF controller. Using AD2015 as an example, Figure 1(A) (top plot) shows that six separate model parameter sets employing six different values of the *FRQ* mRNA Michaelis-Menten kinetic constant (including the original parameter set from Dovzhenok *et. al.* [SR3]) are able to reproduce the *FRQ* mRNA temporal profile. By contrast, as can be seen in as Figure 1(A) (bottom plot), consistent estimates are obtained with the extended S-System model, for which the *FRQ* mRNA activation exponents obtained from different optimisation runs either converge to the base value shown in Table 5, or are unable to reproduce the *FRQ* mRNA profile.

To showcase the effect of inconsistent parameter estimates on AIF controller performance, we apply the controller to restore the loss-of-function of *FRQ* mRNA in the Michaelis-Menten formulation of AD2015, using the original model parameters and also a parameter set with $K_M \sim 0.001$. As shown in Figure 1(B), the controller produces two drastically different behaviors, with a very poor performance observed when $K_M \sim 0.001$. Often, when estimating model parameters from data, we cannot be certain whether an estimated parameter value is the *true* value and a naive design of the AIF controller can therefore lead to poor performance, as we have demonstrated. This issue is mitigated when using the extended S-System models, as they are far less likely to yield inconsistent parameter estimates, and as such are better suited to AIF controller design.

S1.7 Tracking a ramp reference signal

The HYP protein in JD2016 is modeled without a degradation term and from linear control theory, this resembles a pure integrator. Additionally, the monotonically increasing profile of the HYP protein resembles a ramp signal. The overall feedback control configuration can hence be cast as a ramp reference tracking problem, as shown in Figure 16(A). We note that the equations for the steady state error $e(\infty)$, the closed loop transfer function $T(s)$ and all the controller transfer functions presented in this section are standard equations that can be found in any linear control theory textbook (*e.g.* [SR4, SR5]).

As the HYP protein resembles a pure integrator, expressing this in the Laplace domain yields

$$P(s) = \frac{0.18}{s}.$$

Here, the numerator of the process is chosen to be 0.18 as this is the production rate α_{12} of HYP in equations (S2). According to linear control theory, in order to track a ramp reference with zero steady state error, the overall transfer function between the controller $K(s)$ and process $P(s)$ must have at least two integrators and one zero, with the closed loop poles being stable (*i.e.,* lying in the left hand side of the s-plane).

S1.7.1 Tracking with no controller

We first consider the case where there is no controller. The single integrator from the process itself is insufficient to track the ramp reference signal with zero steady state error. To see this, we first note that the steady state error $e(\infty)$ can be calculated using the equation

$$e(\infty) = \frac{sR(s)}{1+\lim_{s\to 0} G(s)}, \tag{S6}$$

where $R(s) = 1/s^2$ is the ramp reference signal and $G(s) = K(s)P(s)$. In the case of no controller, substituting the relevant expressions into equation (S6) gives

$$e(\infty) = \frac{s(1/s^2)}{1+\lim_{s\to 0}(0.18/s)} = \frac{1}{\lim_{s\to 0} s(0.18/s)} = 1/0.18 = 5.56. \tag{S7}$$

A non-zero steady state error thus results, as shown in Figure 16(B).

S1.7.2 Tracking with a pure integral controller

We next consider the case where the controller is a pure integrator $K(s) = 1/s$. The AIF controller with no controller degradation is analagous to this case. The overall transfer function between the controller and the process then has two integrators, with no zero. The closed loop transfer function, is then given by

$$T(s) = \frac{G(s)}{1+G(s)} = \frac{0.18}{s^2+0.18}. \tag{S8}$$

The closed loop poles are obtained by finding the roots of the denominator of the transfer function, *i.e.* $s = \pm j\sqrt{0.18}$, which lie on the imaginary axis of the s-plane. A system with pure imaginary poles is an undamped system, meaning that the response will oscillate continuously (the inverse Laplace transform of $T(s)$ is a sine wave in the time domain). Indeed, as shown in Figure 16(C), the controller is unable to track the ramp reference signal properly, oscillating around it instead. This is precisely the trend we observe in Figure 3(E) in the main text, where the AIF controller oscillates around the HYP reference signal. Note that no steady state error is computed in this case because a system with repeated poles (in this case two poles at the origin) is considered an unstable system, and for such a system the Final Value Theorem does not apply.

S1.7.3 Tracking with a proportional-integral controller

If the overall transfer function of $K(s)P(s)$ has two integrators and a zero, the controller can track the ramp reference signal with zero steady state error. A candidate controller that possesses this characteristic is a proportional-integral (PI) controller. The transfer function of a PI controller is given by

$$K(s) = K_P + \frac{K_I}{s} = \frac{K_P s + K_I}{s}, \tag{S9}$$

where K_P and K_I are the proportional and integral gains respectively. For simplicity, we choose $K_P = K_I = 1$. Substituting the resulting expression into equation (S6), we get

$$e(\infty) = \frac{s(1/s^2)}{1+\lim_{s\to 0}(s+1/s)(0.18/s)} = \frac{1}{\lim_{s\to 0}(s^2+1)(0.18/s)} = 1/\infty = 0. \tag{S10}$$

More importantly, the closed loop transfer function now becomes

$$T(s) = \frac{G(s)}{1+G(s)} = \frac{0.18s+0.18}{s^2+0.18s+0.18}, \tag{S11}$$

and the closed loop poles are $s = -0.09 \pm j0.415$, which lie in the left hand side of the s-plane. As expected, the controller is able to track the ramp reference signal with zero steady state error, albeit following some initial transient behavior, as shown in Figure 16(D) .

S1.7.4 Tracking with a phase lag controller

We now consider the case of a phase lag controller. The transfer function of the controller has the form

$$K(s) = \frac{s+z}{s+p}, \tag{S12}$$

with $|z| > |p|$. As there is no integrator in its transfer function, it follows that a phase lag controller would introduce steady state error when tracking a ramp reference signal. Nevertheless, this controller has the characteristic of improving steady state error and transient effects, at the expense of response speed (see *e.g.* [SR4, SR5]).

A first order transfer function of the form $A/(s+p)$, where A is a constant, is a special case of a phase lag controller with its zero, z, at infinity. With this in mind, we would like to provide an explanation for the substantial improvement in performance obtained with the AIF controller when a non-zero controller degradation ($\gamma_C \neq 0$) is used as compared to when a zero controller degradation ($\gamma_C = 0$) is used (see Figure 4(D) of the main text).

Recall that the AIF controller without degradation resembles a pure integrator. In standard linear control theory (see *e.g.* [SR4, SR5]), introducing a degradation term transforms the transfer function from a pure integrator to a first order transfer function. To illustrate this, we consider the differential equation of a pure integrator, which has the form

$$\frac{dy}{dt} = Ax, \tag{S13}$$

while the differential equation for the integrator with a degradation term is given by

$$\frac{dy}{dt} = Ax - y. \tag{S14}$$

Taking Laplace transforms of both differential equations whilst assuming zero initial conditions results in the following respective transfer functions:

$$\frac{Y(s)}{X(s)} = \frac{A}{s}, \tag{S15}$$

$$\frac{Y(s)}{X(s)} = \frac{A}{s+1}. \tag{S16}$$

With the degradation term being non-zero, we have transformed a pure integrator into a first order transfer function (or a phase lag controller with its zero z at infinity). Thus, switching from zero to non-zero degradation in the AIF controller is analogous to transforming a pure integral controller into a phase lag controller.

If we let $A = \theta_1$, where θ_1 is the AIF controller actuation rate, then this is equivalent to implementing a proportional gain controller, which is an approach that is often used in reducing steady state error (see *e.g.* [SR6]).

In Figure 16(E), we illustrate the effect of varying A, showing that adjusting the proportional gain enables a phase lag controller to reduce the effect of transients and the steady state error, resulting in improved tracking of the ramp reference signal, which is similar to the trend observed in Figure 4(D) of the main text.

S1.8 Model inversion open loop control with feedback

Directly using a synthetically generated transcription factor to regulate affected genes is akin to the *model inversion open loop control* approach [SR7]. Model inversion here refers to designing the actuating signal by inverting the transfer function of the process model, such that applying the actuating signal to the system reproduces the desired output. And open loop control here refers to no feedback being applied. This control strategy is not robust to noise/disturbance and model uncertainty, as these elements can lead to mismatch in the model inversion design. To address the robustness issue, a feedback controller is usually used in combination with the model inversion open loop control.

Using AD2015 as our illustration, we assume that noise/disturbance and model uncertainty leads to the actuating rate of the controller deviating by ±20%. As shown in Figure **??**, implementing the actuating signal (in this case WC-1 transcription factor) designed from model inversion open loop approach alone (Figure **??**(A)) results in poor performance. The inclusion of feedback control (Figure **??**(B)) can improve the performance when the effect of noise reduces the actuating rate, but not when the effect of noise increases the actuating rate.

S1.9 Effect of varying θ_1 for non-unity θ_2

Here, we investigate whether the MSE value can be improved by tuning θ_1 when θ_2 is not unity. We assume that θ_2 is subjected to uncertainty of up to $\pm 30\%$. As can be seen in Figure 14, both with and without the controller degradation term, θ_1 cannot in general be tuned to the same MSE value that is attained when θ_2 is unity. These results suggest that θ_2 should be kept at unity for the best performance in terms of MSE. Nevertheless, in the event that we are not able to set θ_2 to unity due to practical issues, θ_1 can still be fine-tuned to achieve MSE values comparable to those attained when θ_2 is unity.

Supplementary References

(SR1) Foo, M.; Kim, J.; Bates, D. G. (2018) Modelling and control of gene regulatory networks for perturbation mitigation. *IEEE/ACM Trans. Comput. Biol. Bioinform. 16*, 583-595.

(SR2) Foo, M.; Bates, D .G.; Akman, O. E. (2020) A simplified modelling framework facilitates more complex representations of 454 plant circadian clocks. *PLoS Comput. Biol. 16*, e1007671.

(SR3) Dovzhenok, A. A.; Baek, M.; Lim, S.; Hong, C. I. (2015) Mathematical modeling and validation of glucose compensation of the *Neurospora* circadian clock. *Biophys. J.*, *108*, 1830–1839.

(SR4) Nise, N. (2008) *Control Systems Engineering*. 5th ed.; John Wiley and Sons Inc.

(SR5) Franklin, G.; Powell, J.; Emami-Naeini, A. (2018) *Feedback Control of Dynamic Systems*. 8th ed.; Pearson.

(SR6) Li, Y.; Ang, K. H.; Chong, G. C. Y. (2006) PID control system analysis and design. *IEEE Control Syst. Lett. 26*, 32-41.

(SR7) Foo, M.; Kim, J.; Sawlekar, R.; Bates, D.G. (2017) Design of an embedded inverse-feedforward biomolecular tracking controller for enzymatic reaction processes. *Comput. Chem. Eng.*, *99*, 145–157.

(SR8) Locke, J. C. W.; Millar, A. J.; Turner, M. S. (2005) Modelling genetic networks with noisy and varied experimental data: the circadian clock in *Arabidopsis thaliana*. *J. Theor. Biol.*, *234*, 383–392.

(SR9) De Caluwe, J.; Xiao, Q.; Hermans, C.; Verbruggen, N.; Leloup, J. L.; Gonze, D. (2016) A compact model for the complex plant circadian clock. *Front. Plant Sci.*, *7*, 74.

(SR10) Becker-Weimann, S.; Wolf, J.; Herzel, H.; Kramer, A. (2004) Modeling feedback loops of the mammalian circadian oscillator. *Biophys. J.*, *87*, 3023–3034.

(SR11) Ueda, H.; Hagiwara, M.; Kitano, H. (2001) Robust oscillations within the interlocked feedback model of *Drosophila* circadian rhythm. *J. Theor. Biol.*, *210*, 401–406.

Supplementary Tables

Supplemental Table 1. Variables used in the equations for the plant, fruitfly and fungal clock models. The notation employed here matches that used in the original references.

i	Gene	i	Gene
L	LHY/CCA1	FW	FRQ/WC-1
T	TOC1	$CSP1$	CSP-1
CL	CCA1/LHY	FRQ	FRQ
$P97$	PRR9/PRR7	CC	CLK/CYC
$P51$	PRR5/TOC1	$PTCC$	PER/TIM/CLK/CYC
EL	ELF4/LUX	PER	PER
PIF	PIF4/PIF5	TIM	TIM
$PER2CRY$	PER2/CRY	PT	PER/TIM
$BMAL1$	BMAL1	PCC	PER/CLK/CYC
$WC1$	WC-1	CLK	CLK

Supplemental Table 2. Parameter values for JL2005 using the extended S-System model. The model parameters are estimated from the synthetic time series generated from [SR8].

JL2005					
Parameter	Value	Parameter	Value	Parameter	Value
α_1	1.4361	$g_{1,1}$	1.3276	$\beta_{1,1}$	1.2875
$\gamma_{1,1}$	4.7463	α_2	0.4074	$\gamma_{2,1}$	2.0764
$\beta_{2,1}$	1.7443	α_3	0.9147	$\gamma_{3,1}$	1.7344
$\beta_{3,1}$	1.4088	α_4	0.0484	$\gamma_{4,1}$	−1.4874
$\beta_{4,1}$	1.5163	α_5	2.3917	$\gamma_{5,1}$	1.9354
$\beta_{5,1}$	0.2075	α_6	0.0378	$\gamma_{6,1}$	1.1309
$\beta_{6,1}$	0.0268	$\beta_{7,1}$	0.4886	$\gamma_{7,1}$	0.4080
$\gamma_{7,2}$	−1.5690	—	—	—	—

Supplemental Table 3. Parameter values for JD2016 using the extended S-System model. The model parameters are estimated from the synthetic time series generated from [SR9].

JD2016					
Parameter	Value	Parameter	Value	Parameter	Value
α_1	0.7229	$g_{1,1}$	−1.2502	$g_{1,2}$	−0.0421
$\beta_{1,1}$	0.7524	$\gamma_{1,1}$	−0.9717	α_2	0.8063
$\beta_{2,1}$	0.7489	$\gamma_{2,1}$	0.7469	α_3	0.0264
$g_{3,1}$	0.1241	$g_{3,2}$	−2.3687	$g_{3,3}$	−0.1056
$\beta_{3,1}$	0.3539	$\gamma_{3,1}$	1.5597	α_4	1.0733
$\beta_{4,1}$	0.2969	$\gamma_{4,1}$	−0.0506	α_5	0.3836
$g_{5,1}$	−0.7167	$g_{5,1}$	0.1736	$\beta_{5,1}$	1.013
α_6	0.552	$\beta_{6,1}$	0.4167	$\gamma_{6,1}$	−0.2793
$\gamma_{7,1}$	0.011	$g_{7,1}$	−1.8819	$g_{7,2}$	−1.3932
$g_{7,3}$	−0.0172	$\beta_{7,1}$	0.4814	α_8	1.0696
$\beta_{8,1}$	1.3282	$\gamma_{8,1}$	0.9105	α_9	0.3482
$\beta_{9,1}$	0.3675	$\gamma_{9,1}$	−0.3482	$\gamma_{9,2}$	−0.2732
α_{10}	0.0363	$g_{10,1}$	−0.2808	$\beta_{10,1}$	0.1971
α_{11}	0.3097	$\beta_{11,1}$	0.1832	$\gamma_{11,1}$	−2.0456

α_{12}	0.174	$g_{12,1}$	1.1843	—	—

Supplemental Table 4. Parameter values for SB2004 using the extended S-System model. The model parameters are estimated from the synthetic time series generated from [SR10].

SB2004					
Parameter	Value	Parameter	Value	Parameter	Value
α_1	0.1496	$g_{1,1}$	−5.5475	$g_{1,2}$	0.0451
$\beta_{1,1}$	0.1500	α_2	0.3215	$g_{2,1}$	1.7465
$\beta_{2,1}$	0.2706	α_3	0.1761	$g_{3,1}$	1.3147
$\beta_{3,1}$	0.1032	α_4	0.8097	$g_{4,1}$	2.0570
$\beta_{4,1}$	1.5816	α_5	0.2024	$g_{5,1}$	0.9527
$\beta_{5,1}$	0.3472	α_6	0.3850	$g_{6,1}$	1.0573
$\beta_{6,1}$	0.2212	α_7	0.1168	$g_{7,1}$	1.0321
$\beta_{7,1}$	0.1206	—	—	—	—

Supplemental Table 5. Parameter values for AD2015 using the extended S-System model. The model parameters are estimated from the synthetic time series generated from [SR3].

AD2015					
Parameter	Value	Parameter	Value	Parameter	Value
α_1	0.8057	$g_{1,1}$	3.9937	$\beta_{1,1}$	0.2749
α_2	1.5144	$\beta_{2,1}$	0.251	α_3	0.0507
$\beta_{3,1}$	0.2867	$\beta_{3,2}$	48.8696	$\beta_{3,3}$	−1.0255
α_4	0.438	$g_{4,1}$	−0.1273	$\beta_{4,1}$	0.118
α_5	0.0664	$g_{5,1}$	1.0938	$g_{5,2}$	0.2042
$\beta_{5,1}$	64.3947	α_6	42.0093	$\beta_{6,1}$	0.0148
$\beta_{6,2}$	51.0546	$\beta_{6,3}$	−0.9058	α_7	52.0211
$\beta_{7,1}$	6.3082	α_8	0.063	$g_{8,1}$	0.9262
$g_{8,2}$	−0.1793	$\beta_{8,1}$	1.0502	α_9	52.412
$\beta_{9,1}$	1.4815	—	—	—	—

Supplemental Table 6. Parameter values for HU2001 using the extended S-System model. The model parameters are estimated from the synthetic time series generated from [SR11].

HU2001					
Parameter	Value	Parameter	Value	Parameter	Value
α_1	0.4618	$g_{1,1}$	1.2751	$g_{1,2}$	−0.0135
$\beta_{1,1}$	0.5238	α_2	0.4389	$g_{2,1}$	1.0030
$\beta_{2,1}$	0.7035	$\beta_{2,2}$	1.3918	$\beta_{2,3}$	−1.7507
α_3	0.3766	$g_{3,1}$	1.4944	$g_{3,2}$	−0.0194
$\beta_{4,1}$	0.4856	$\alpha_{4,1}$	0.3440	$g_{4,1}$	1.0101
$\beta_{4,1}$	0.5299	$\beta_{4,2}$	1.3262	$\beta_{4,3}$	−1.6196
α_5	1.4410	$g_{5,1}$	1.4995	$g_{5,2}$	0.8609
$\beta_{5,1}$	2.1687	α_6	0.2821	$g_{6,1}$	1.2101
$\beta_{6,1}$	0.2414	α_7	0.2965	$g_{7,1}$	−1.7563
$g_{7,2}$	0.0024	$\beta_{7,1}$	0.1512	α_8	0.1768
$g_{8,1}$	1.6382	$\beta_{8,1}$	2.2927	$\beta_{8,2}$	2.3236
α_9	1.4296	$g_{9,1}$	1.2849	$\beta_{9,1}$	2.5544
α_{10}	0.1794	$g_{10,1}$	1.7833	$\beta_{10,1}$	0.16931

Supplementary Figures

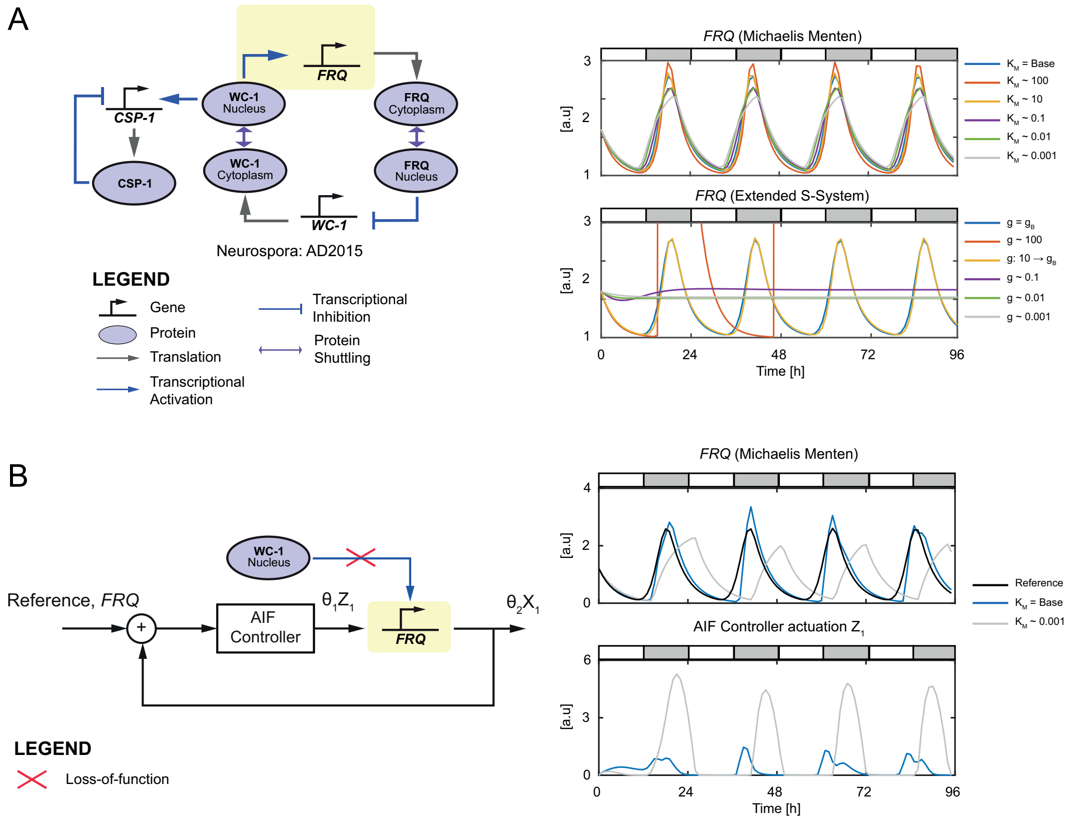

Supplemental Figure 1. Illustrative example of modeling and AIF control – the *Neurospora* circadian clock model AD2015. (A) *FRQ* mRNA is modeled using Michaelis-Menten and extended S-System model structures. For the Michaelis-Menten formulation, parameter sets containing six different Michaelis-Menten constant values, including the base parameter values used in ref.[26], are able to reproduce the *FRQ* mRNA profile. For the extended S-System formulation, the activation exponent g either converges to the base value (Table 5) or yields a parameter set that is unable to reproduce the *FRQ* mRNA profile. (B) The performance of the same AIF controller applied to the Michaelis-Menten formulation, but with two different parameter sets. The white and grey bars correspond to light and subjective light intervals, respectively. The notation $\sim$ indicates that the Michaelis-Menten constant is close to the stated value, while the notation $\rightarrow$ indicates that the estimated exponent converges to the base value.

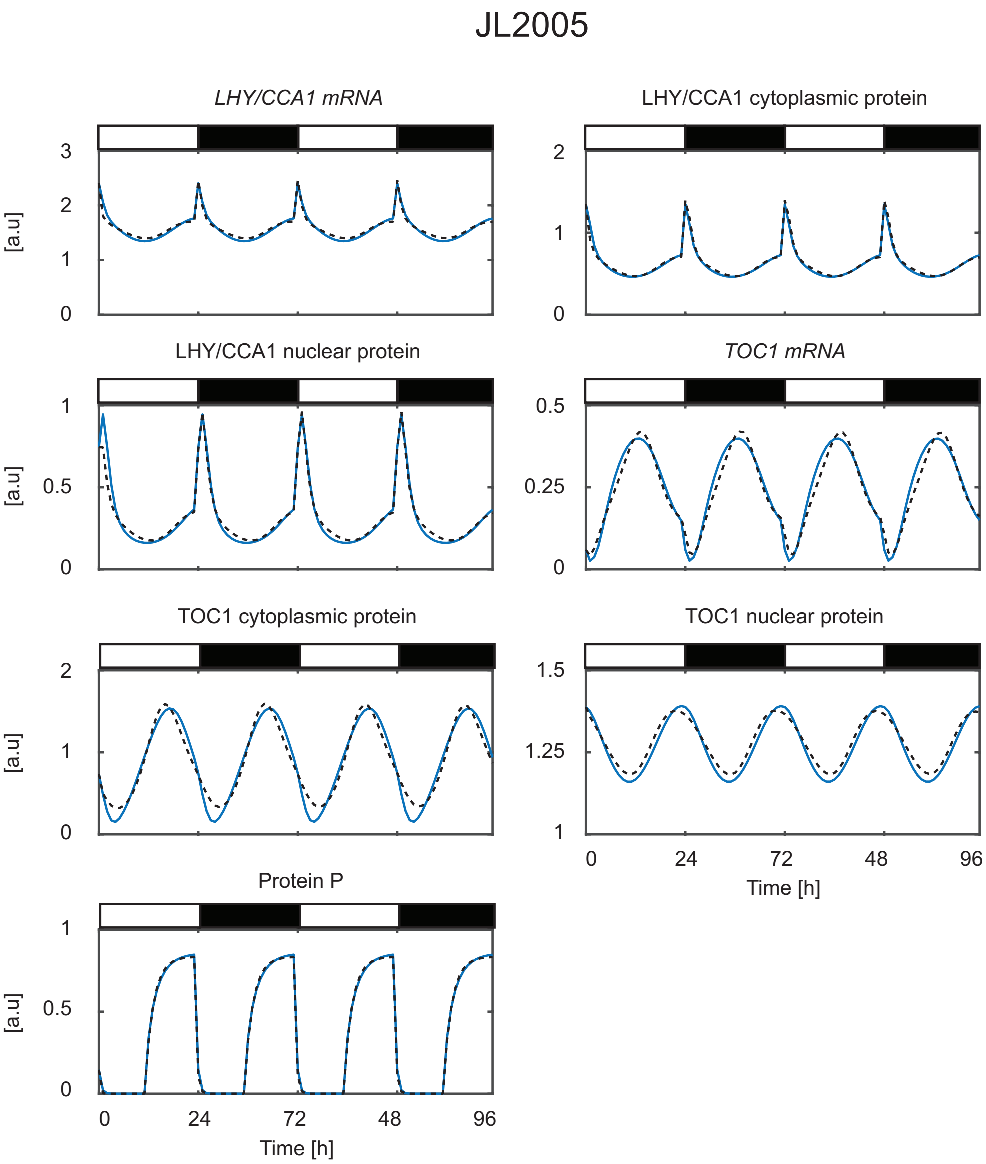

Supplemental Figure 2. **Time series comparison in JL2005.** Solid blue lines: Time series generated in simulated 12L:12D light-dark cycles using the Michaelis-Menten model structure. Dashed black lines: Corresponding time series obtained with the extended S-System formulation. White and black rectangular boxes at the top of the figure correspond to light and dark intervals, respectively.

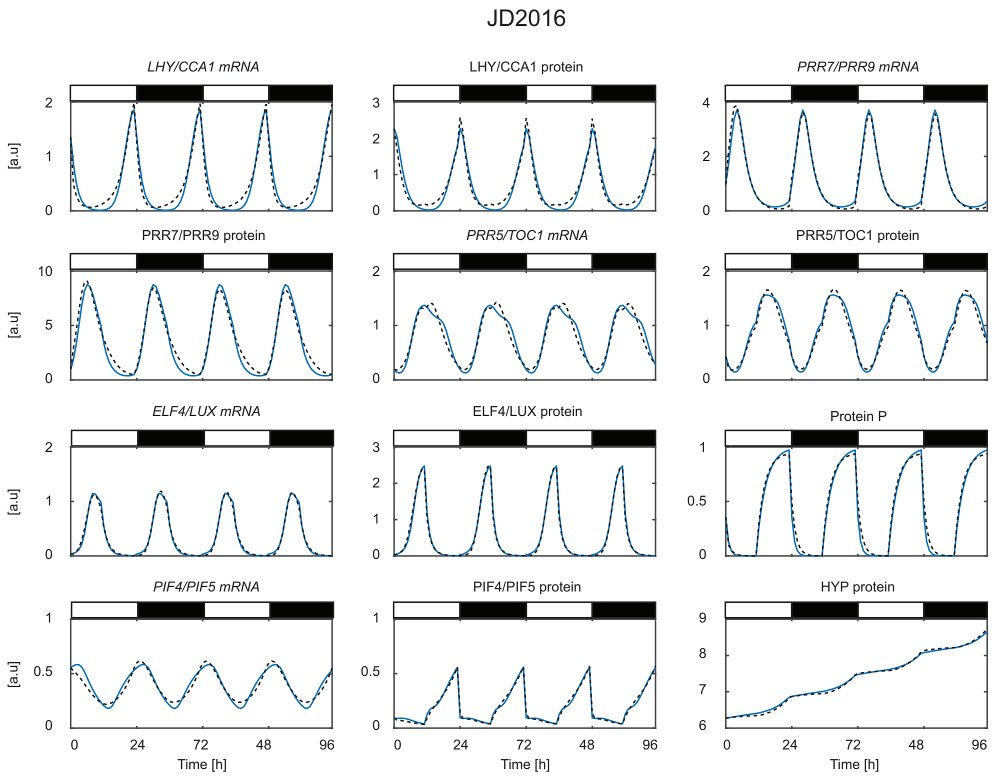

Supplemental Figure 3. **Time series comparison in JD2016.** Solid blue lines: Time series generated in simulated 12L:12D light-dark cycles using the Michaelis-Menten model structure. Dashed black lines: Corresponding time series obtained with the extended S-System formulation. White and black rectangular boxes at the top of the figure correspond to light and dark intervals, respectively.

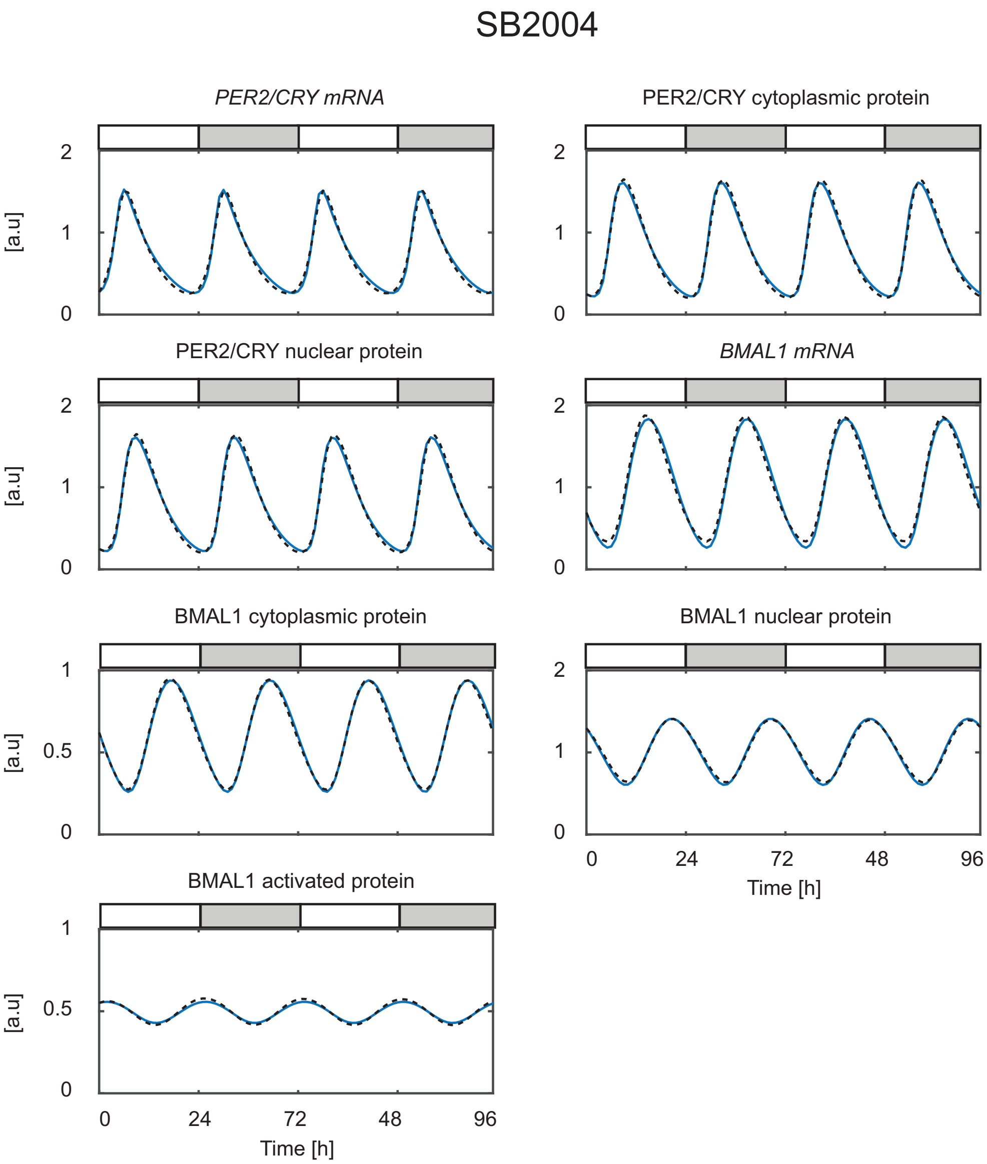

Supplemental Figure 4. Time series comparison in SB2004. Solid blue lines: Time series generated in simulated 12L:12D light-dark cycles using the Michaelis-Menten model structure. Dashed black lines: Corresponding time series obtained with the extended S-System formulation. White and gray rectangular boxes at the top of the figures correspond to light and subjective dark intervals, respectively.

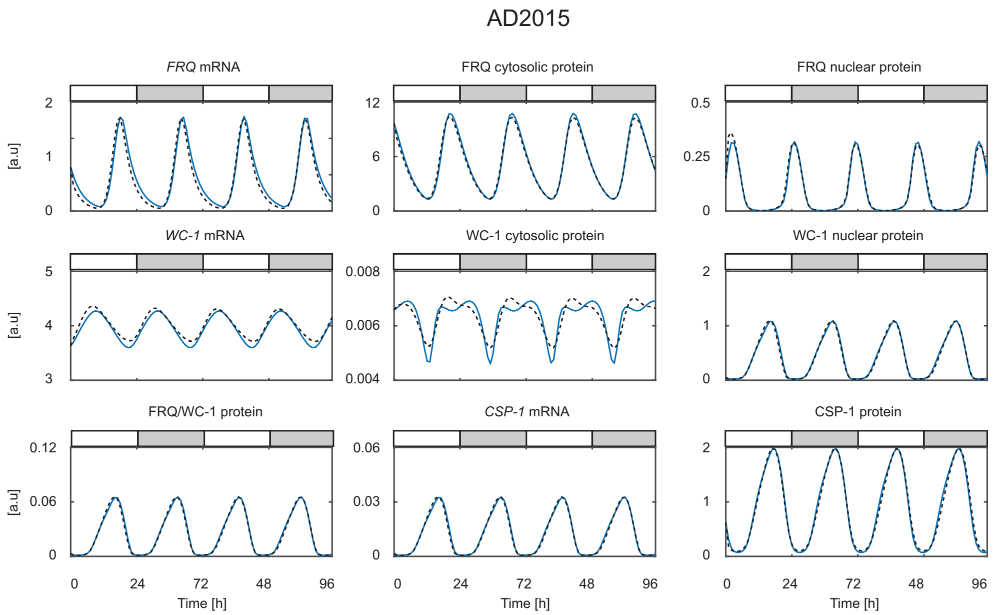

Supplemental Figure 5. Time series comparison in AD2015. Solid blue lines: Time series generated in simulated 12L:12D light-dark cycles using the Michaelis-Menten model structure. Dashed black lines: Corresponding time series obtained with the extended S-System formulation. White and gray rectangular boxes at the top of the figures correspond to light and subjective dark intervals, respectively.

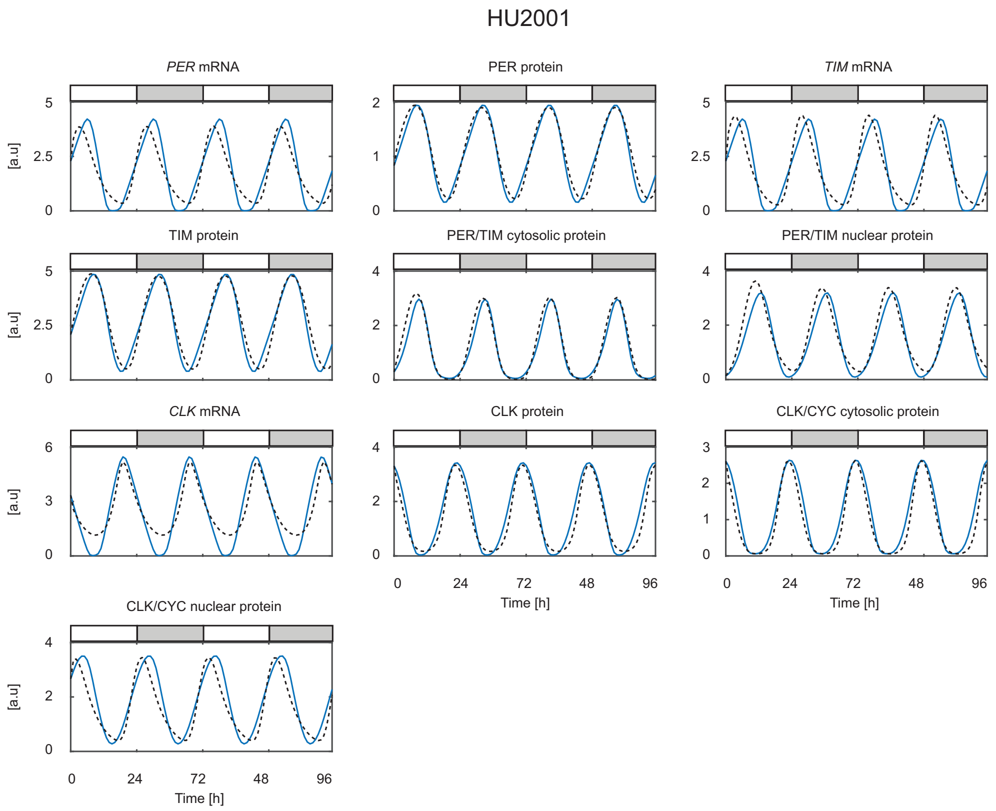

Supplemental Figure 6. Time series comparison in HU2001. Solid blue lines: Time series generated in simulated 12L:12D light-dark cycles using the Michaelis-Menten model structure. Dashed black lines: Corresponding time series obtained with the extended S-System formulation. White and gray rectangular boxes at the top of the figures correspond to light and subjective dark intervals respectively.

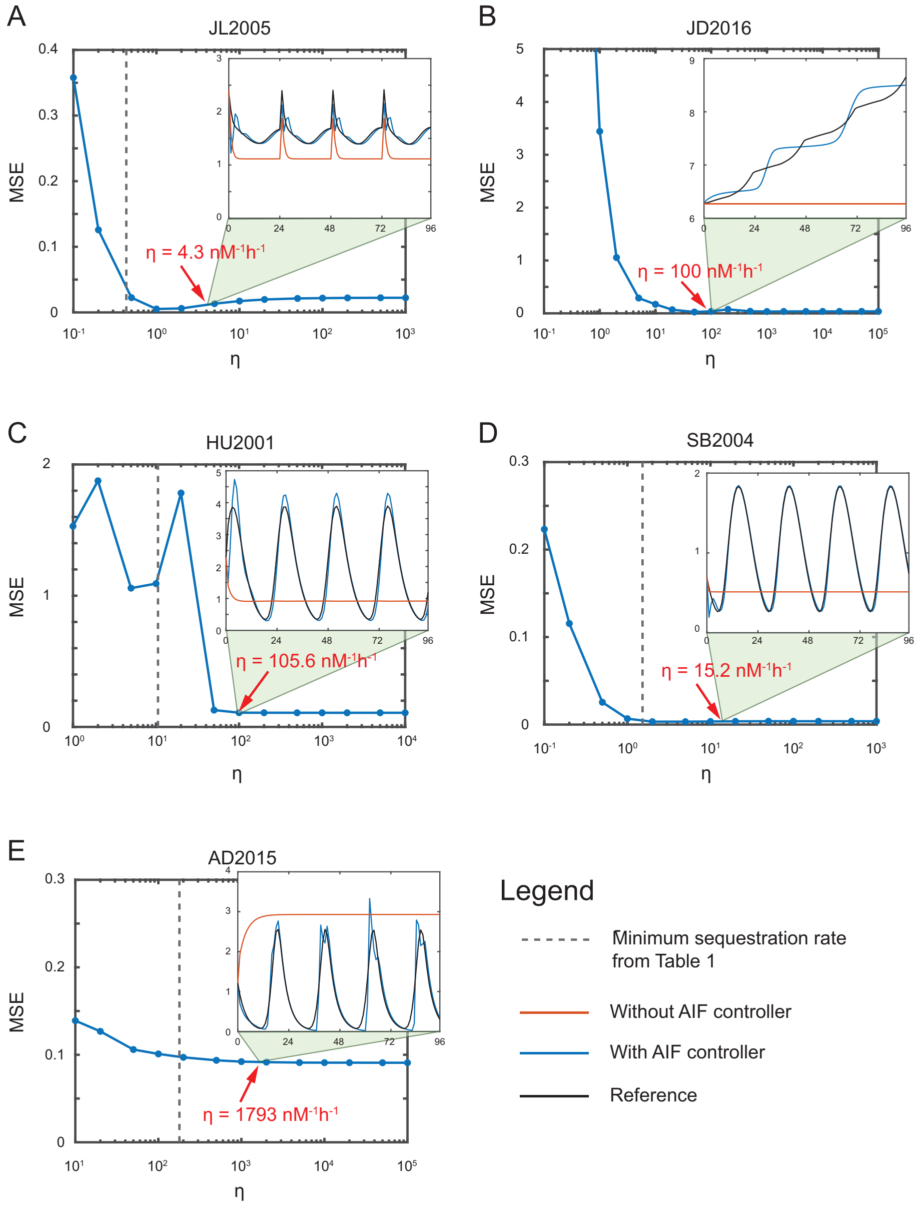

Supplemental Figure 7. Effect of varying the sequestration rate η on the mean square error (MSE) between the reference profile and the process output when the AIF controller is applied with $\gamma_C = 0$ h^{-1} and $\theta_1 = \theta_2 = 1$ h^{-1}. (A) Plant clock, JL2005. (B) Plant clock, JD2016. (C) Insect clock, HU2001. (D) Mammalian clock, SB2004. (E) Fungal clock, AD2015. The inset figures in (A)-(E) show the time series profiles for the reference profile and process output, with and without the use of the AIF controller.

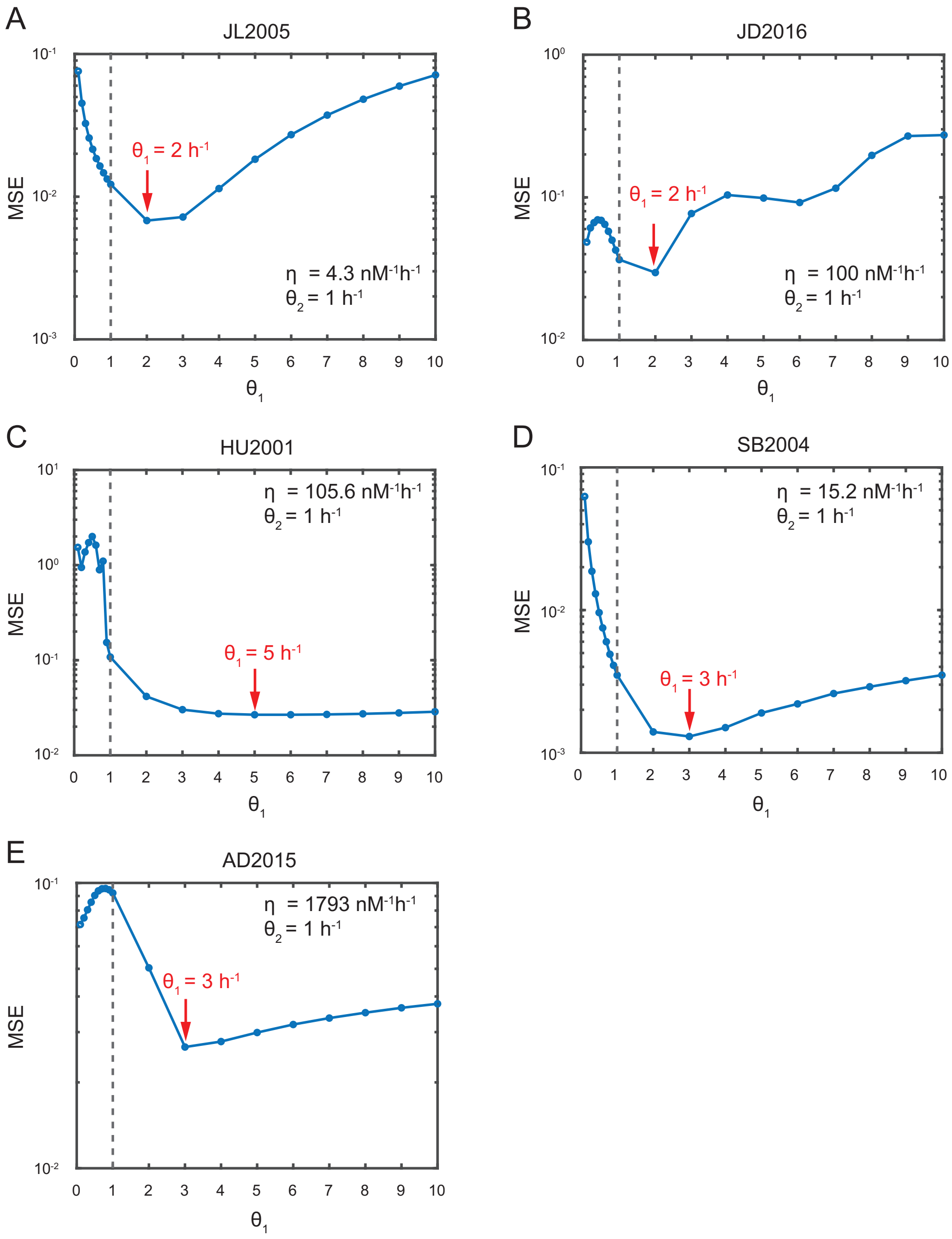

Supplemental Figure 8. Effect of varying θ_1 on the mean square error (MSE) between the reference profile and the process output when the AIF controller is applied with $\gamma_C = 0$ h^{-1}, $\eta = 10\bar{\eta}$ and $\theta_2 = 1$ h^{-1}. (A) Plant clock, JL2005. (B) Plant clock, JD2016. (C) Insect clock, HU2001. (D) Mammalian clock, SB2004. (E) Fungal clock, AD2015. In each panel, the red arrow represents the θ_1 value that produces the smallest MSE.

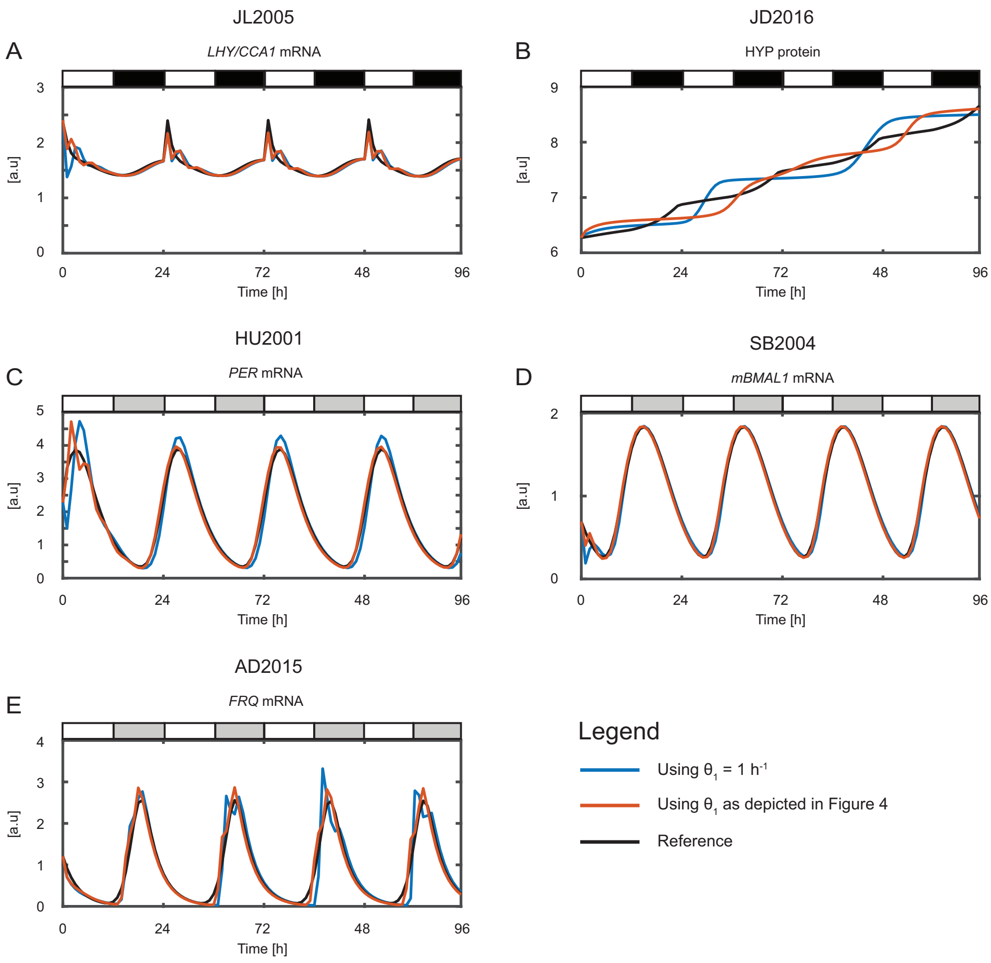

Supplemental Figure 9. Comparison of the time series profiles obtained using the AIF controller for different values of θ_1 with $\gamma_C = 0$ h^{-1}, $\eta = 10\bar{\eta}$ and $\theta_2 = 1$ h^{-1}. (A) Plant clock, JL2005. (B) Plant clock, JD2016. (C) Insect clock, HU2001. (D) Mammalian clock, SB2004. (E) Fungal clock, AD2015. Black lines: reference signals, Blue lines: controller outputs obtained with $\theta_1 = 1$ h^{-1}. Red lines: controller outputs obtained with the optimal θ_1 values indicated in Figure 8. White, black and grey rectangular boxes at the top of the figure correspond to light, dark and subjective dark intervals respectively.

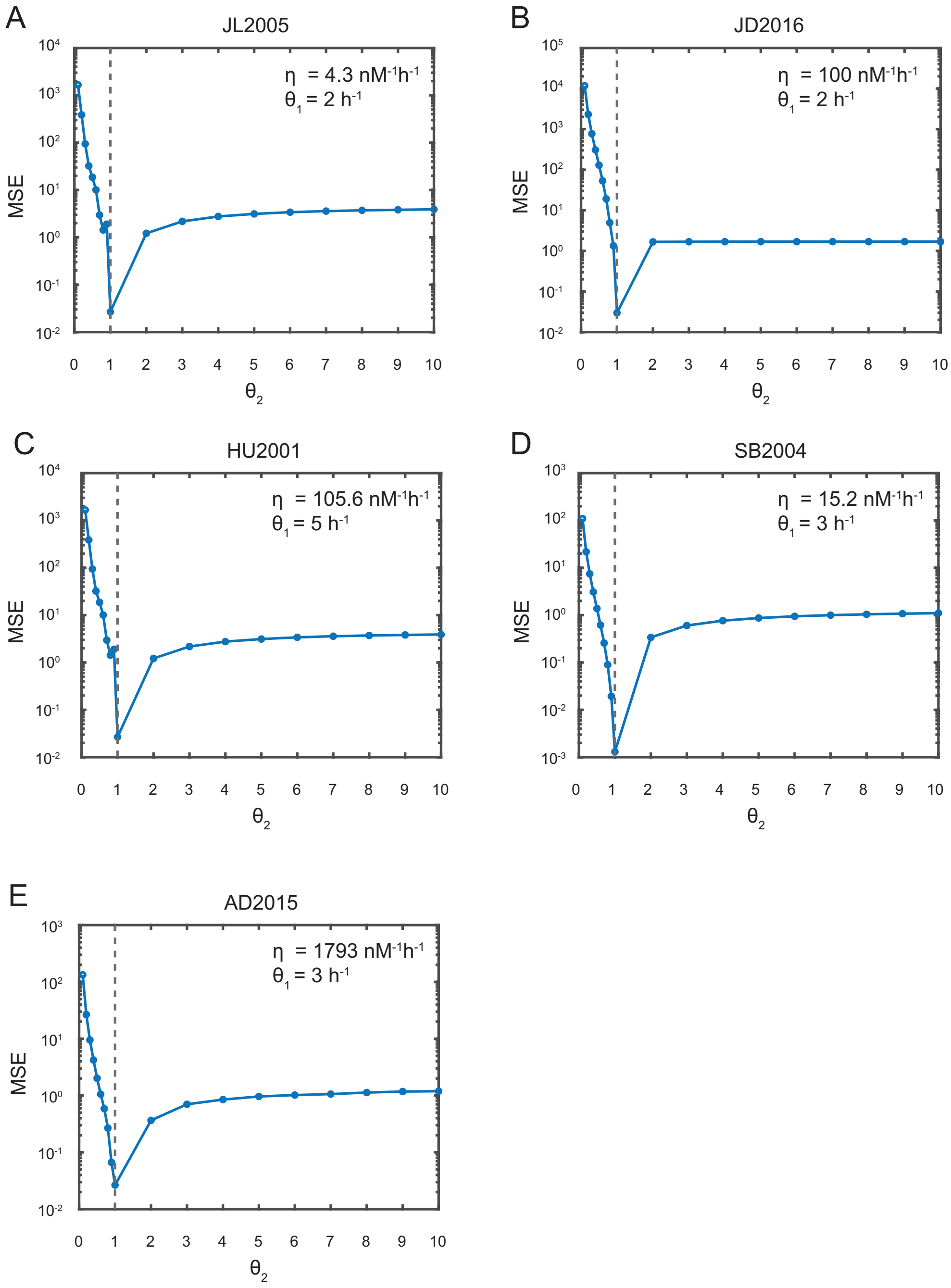

Supplemental Figure 10. Effect of varying θ_2 on the mean square error (MSE) between the reference profile and the process output when the AIF controller is applied with $\gamma_C = 0$ h^{-1} and $\eta = 10\bar{\eta}$. (A) Plant clock, JL2005. (B) Plant clock, JD2016. (C) Insect clock, HU2001. (D) Mammalian clock, SB2004. (E) Fungal clock, AD2015. In each set of simulations, we set θ_1 to the value indicated by the red arrow in the corresponding panel of Figure 8 of the main text.

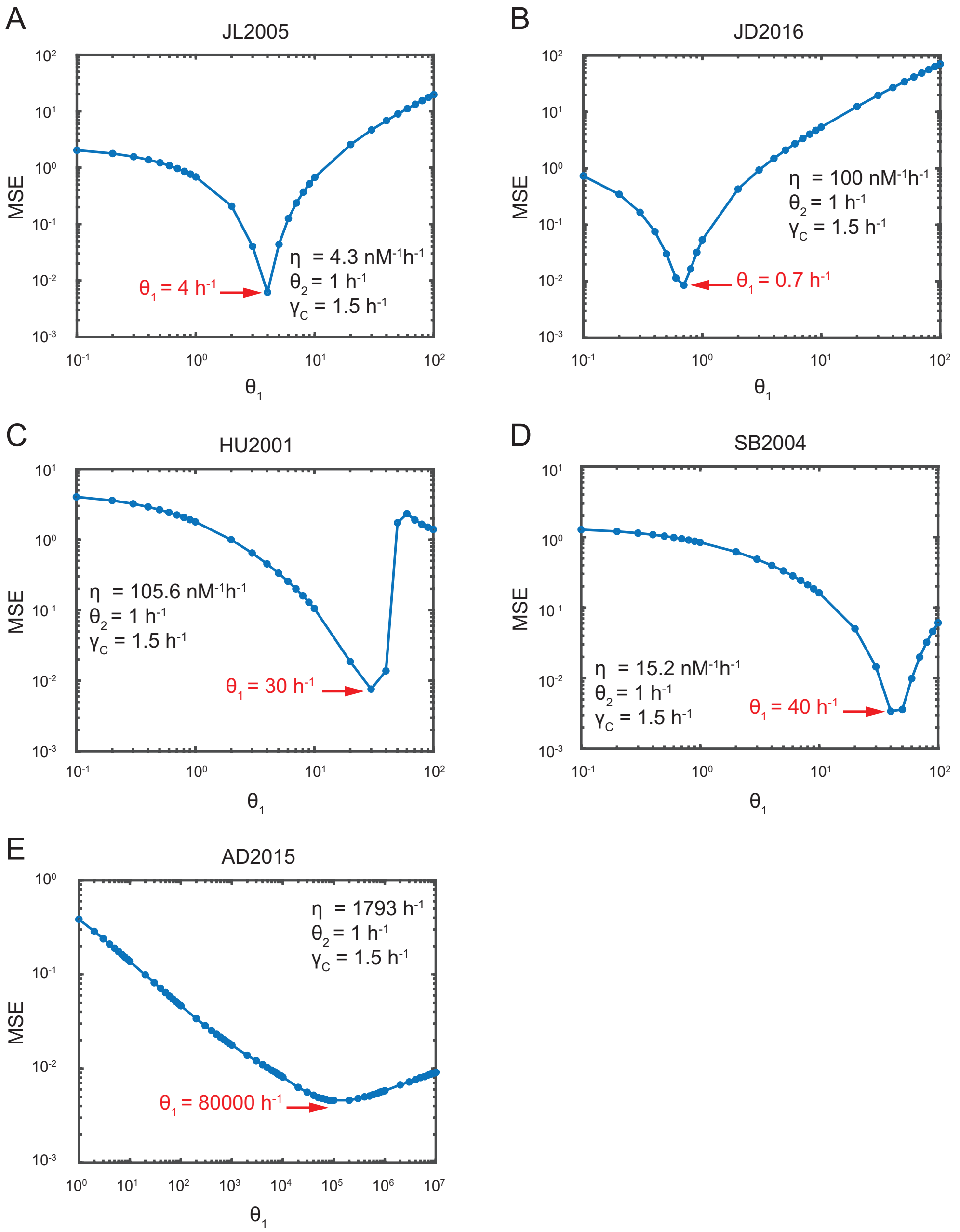

Supplemental Figure 11. Effect of varying θ_1 on the mean square error (MSE) between the reference profile and the process output when AIF controller is applied with $\gamma_C = 1.5$ h^{-1}, $\eta = 10\bar{\eta}$ and $\theta_2 = 1$ h^{-1}. (A) Plant clock, JL2005. (B) Plant clock, JD2016. (C) Insect clock, HU2001. (D) Mammalian clock, SB2004. (E) Fungal clock, AD2015. The θ_1 values yielding the smallest MSE value are indicated by the red arrow in each panel.

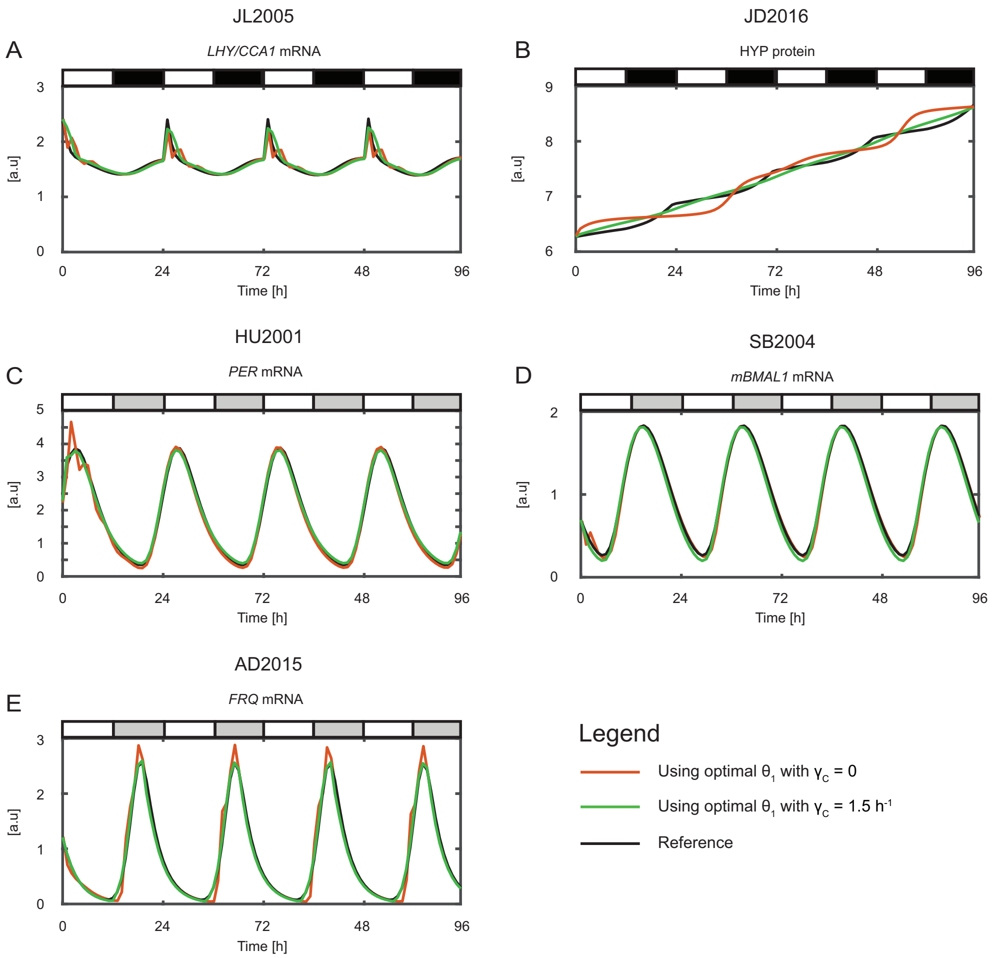

Supplemental Figure 12. Comparison of the time series profiles obtained using the AIF controller for different values of θ_1 with $\gamma_C = 1.5$ h^{-1}, $\eta = 10\bar{\eta}$ and $\theta_2 = 1$ h^{-1}. (A) Plant clock, JL2005. (B) Plant clock, JD2016. (C) Insect clock, HU2001. (D) Mammalian clock, SB2004. (E) Fungal clock, AD2015. Black lines: reference signals. Green lines: controller outputs generated with optimal θ_1 values taken from Figure 11. Red lines: controller outputs generated with optimal θ_1 values taken from Figure 8. White, black and grey rectangular boxes at the top of the figure correspond to light, dark and subjective dark intervals respectively.

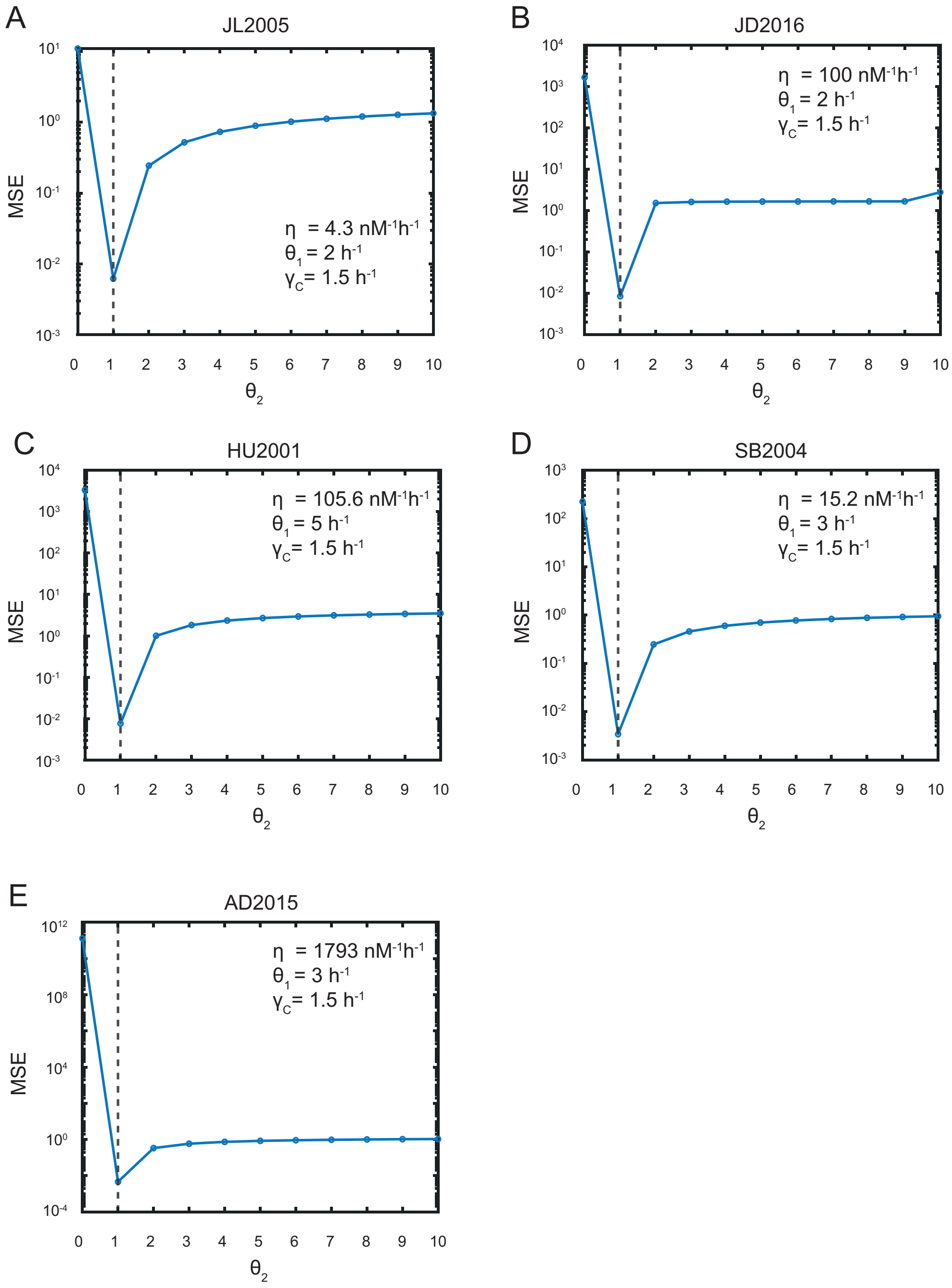

Supplemental Figure 13. Effect of varying θ_2 on the mean square error (MSE) between the reference profile and the process output when the AIF controller is applied with $\gamma_C = 1.5$ h^{-1} and $\eta = 10\bar{\eta}$. (A) Plant clock, JL2005. (B) Plant clock, JD2016. (C) Insect clock, HU2001. (D) Mammalian clock, SB2004. (E) Fungal clock, AD2015. In each set of simulations, we set θ_1 to the value indicated by the red arrow in the corresponding panel of Figure 11 of the main text.

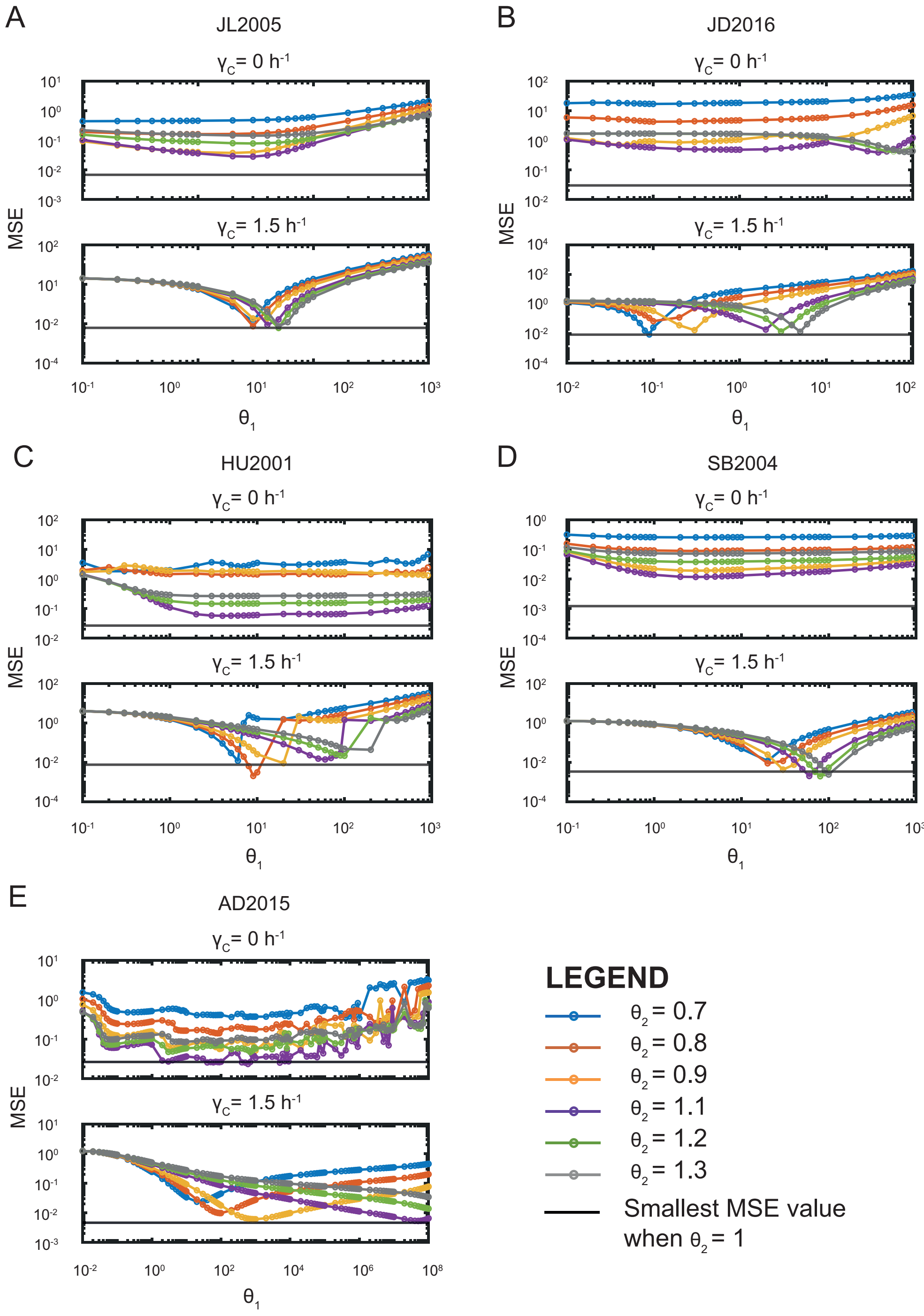

Supplemental Figure 14. Effect of fine-tuning θ_1 when θ_2 is non-unity for all circadian clock models. (A) Plant clock, JL2005. (B) Plant clock, JD2016. (C) Insect clock, HU2001. (D) Mammalian clock, SB2004. (E) Fungal clock, AD2015. In each set of simulations, the MSE value when θ_2 is unity is shown by the black solid lines.

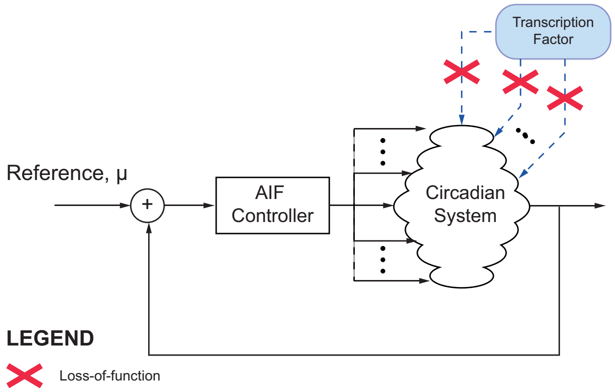

Supplemental Figure 15. AIF control for complex clock network. Possible strategy for redesigning the AIF controller to restore function in a transcription factor that has widespread repercussions in the overall clock network.

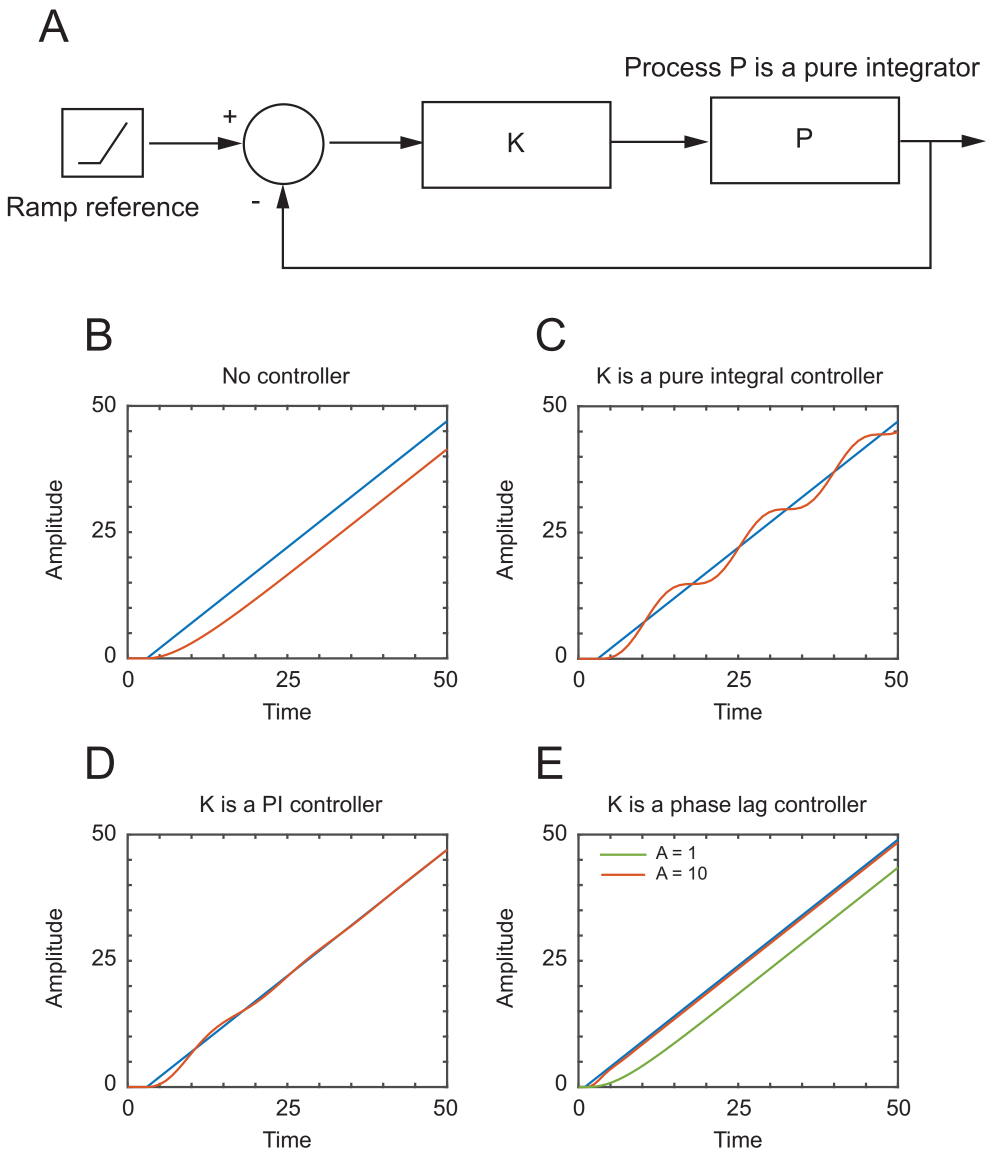

Supplemental Figure 16. **Tracking a ramp reference signal with different feedback controllers.** (A) Control configuration with feedback. (B) Ramp response when there is no controller. (C) Ramp response when K is a pure integral controller. (D) Ramp response when K is a proportional-integral (PI) controller. (E) Ramp response when K is a phase lag controller, where the controller has one zero and one pole with the value of the zero larger than that of the pole. In panels (B) to (E), the blue solid line is the reference (ramp) signal and the red solid line is the output response. In panel (E), the green solid line indicates the output response obtained with a different A value.
